# The Role of Gut Microbiota and Their Derived Metabolites in Chemotherapy‐Induced Nausea and Vomiting in Ovarian Cancer

**DOI:** 10.1002/cam4.71752

**Published:** 2026-04-02

**Authors:** Shuiling Zu, Xiaoyan Yu, Jihong Song, Yu Xiao, Huan Yi, Hong Li

**Affiliations:** ^1^ Department of Gynecology, Fujian Maternity and Child Health Hospital, College of Clinical Medicine for Obstetrics & Gynecology and Pediatrics Fujian Medical University Fuzhou China; ^2^ School of Nursing Fujian Medical University Fuzhou China

**Keywords:** chemotherapy, gut microbiota, nausea, ovarian cancer, vomiting

## Abstract

**Objective:**

This study aimed to investigate the relationship between gut microbiota and chemotherapy‐induced nausea and vomiting (CINV) in patients with ovarian cancer undergoing platinum‐based chemotherapy (carboplatin or cisplatin combined with paclitaxel).

**Methods:**

Clinical data and fecal samples were collected from patients with ovarian cancer after admission but prior to the initiation of their first chemotherapy cycle. Patients were divided into the CINV (*n* = 25) and non‐CINV (*n* = 25) groups on the basis of symptoms occurring after chemotherapy. No additional samples were collected during chemotherapy. Integrated metagenomic sequencing and untargeted metabolomic profiling identified CINV‐associated microbial taxa and metabolites. Additionally, fecal microbiota transplantation (FMT) in SD rats validated causal links between gut dysbiosis and CINV pathogenesis.

**Results:**

*Bacteroides caccae* , Corynebacteriales, and *Corynebacterium* were significantly enriched in the CINV group. KEGG enrichment revealed upregulated pathways in CINV, including focal adhesion, lysosome function, and eukaryotic cellular communities. Metabolomic analysis identified 19 significantly increased metabolites in the fecal samples of CINV patients versus 10 in non‐CINV controls. KEGG enrichment revealed that the pentose phosphate pathway, glutathione metabolism, and lipoic acid metabolism were significantly implicated in CINV pathogenesis. Multi‐omics integration revealed *Bacteroides* sp. A1C1 strongly correlated with hesperetin, arbutin, orciprenaline, and myristolic acid. In rats, cisplatin‐induced CINV models showed higher kaolin consumption versus controls (*p* < 0.05). FMT from non‐CINV donors reduced kaolin consumption in cisplatin‐treated rats (*p* < 0.05). The expression of 5‐HT3R, NK1R, and NK2R in the medulla oblongata and colon was significantly increased in the cisplatin model group (*p* < 0.05) and partially reversed by non‐CINV FMT (*p* < 0.05).

**Conclusions:**

Gut microbiota dysbiosis directly contributes to CINV pathogenesis. *Bacteroides* sp. A1C1 and its putatively identified metabolites (hesperetin, arbutin, orciprenaline, and myristolic acid) represent potential diagnostic biomarkers for CINV.

## 1 Introduction

Ovarian cancer is a significant global health concern, with an estimated 313,959 new cases and 207,252 deaths worldwide in 2020 [1]. The vast majority of ovarian cancer patients require chemotherapy in addition to surgery. However, chemotherapy‐induced nausea and vomiting (CINV) is the most common complication in patients undergoing chemotherapy. Its incidence is as high as 65%–85%, and it is a major factor seriously affecting patients' quality of life and treatment compliance [2, 3]. In addition, severe vomiting can lead to electrolyte imbalance, difficulty eating, nutritional deficiencies, and ultimately, a decline in immune function [4, 5].

Currently, the combination of 5‐hydroxytryptamine type 3 receptor (5‐HT3R) antagonists and neurokinin‐1 receptor (NK1R) antagonists can significantly inhibit nausea and reduce the rates of both acute and delayed vomiting in cancer patients receiving chemotherapy [6, 7, 8, 9]. However, many patients still experience nausea and delayed vomiting, in addition to common adverse reactions such as headache, diarrhea, constipation, hiccups, and fatigue [10, 11, 12]. In addition, the use of second‐generation 5‐HT3R and NK1R antagonists for CINV prevention is expensive for many patients worldwide. Further research is needed to identify more effective and accessible treatment strategies.

Over the past 25 years, the pathogenesis of CINV has not been fully elucidated, and its neurotransmitter mechanisms remain a key research focus [13, 14]. Chemotherapy drug‐generated free radicals can disrupt the intestinal barrier, causing jejunal chromaffin cells to release serotonin [13, 15]. Serotonin binds to 5‐HT3R on the enteric vagus nerve, triggering the vomiting reflex via the solitary tract nucleus and chemoreceptor trigger zone in the central nervous system [15]. In recent years, advances in next‐generation sequencing have revealed the involvement of the gut microbiota in CINV. Chemotherapy drugs can impact the gut‐brain axis by altering the composition and function of the gut microbiota [16]. Gut microbiota dysbiosis damages the intestinal wall and further stimulates intestinal chromaffin cells, inducing serotonin release [17]. Gut microbiota dysbiosis can also activate inflammatory cells, including macrophages and T lymphocytes, thereby inducing the release of pro‐inflammatory cytokines and chemokines [18, 19]. These findings suggest that the gut microbiota may contribute directly or indirectly to CINV. However, the specific role of the gut microbiota in CINV among ovarian cancer patients remains poorly understood.

The aim of this study was to investigate the role of the gut microbiota in ovarian cancer patients experiencing CINV. Additionally, we aimed to identify related gut metagenomic features and metabolites.

## 2 Methods

### 2.1 Patient Samples

Fecal samples and clinical data were collected from patients who underwent chemotherapy for ovarian cancer at our institution between December 1, 2023, and December 31, 2024. Inclusion criteria were: (1) age ≥ 18 years; (2) clinical stage IC‐IVA ovarian cancer according to the 2014 FIGO staging system; (3) receipt of platinum‐based chemotherapy (carboplatin or cisplatin combined with paclitaxel); (4) absence of infectious diseases or serious cardiac, hepatic, or hematopoietic dysfunction within 2 months prior to enrollment; and (5) no use of antibiotics, probiotics, or other medications known to affect gut microbiota within 1 month prior to sample collection. Exclusion criteria were: (1) postoperative follow‐up duration less than 72 h. Finally, 25 patients with CINV and 25 without CINV were enrolled.

### 2.2 Data and Samples Collection

Fecal samples and clinical data were collected after admission but prior to the initiation of chemotherapy. Collected clinical data included age, height, weight, tumor type, tumor stage, chemotherapy regimen, and symptoms of nausea and vomiting. The primary endpoint was the occurrence of CINV following chemotherapy.

The National Cancer Institute Common Terminology Criteria for Adverse Events (NCI‐CTCAE), version 5.0, defines nausea as a disorder characterized by a queasy sensation and/or urge to vomit. Grading is as follows: Grade 1: Loss of appetite without alteration in eating habits; Grade 2: Oral intake decreased without significant weight loss, dehydration, or malnutrition; Grade 3: Inadequate oral caloric or fluid intake; tube feeding, total parenteral nutrition (TPN), or hospitalization indicated. Vomiting is defined as a disorder characterized by the reflexive act of expelling gastric contents through the mouth. Grading: Grade 1: Intervention not indicated; Grade 2: Outpatient intravenous hydration; medical intervention indicated; Grade 3: Tube feeding, TPN, or hospitalization indicated; Grade 4: Life‐threatening consequences; Grade 5: Death [20].

Fecal samples were collected from all participants. (1) The middle portion of the stool was collected into a sterile container, ensuring no urine contamination. (2) Fecal samples (at least 1 g) were collected using a sterile spoon, placed in a cryovial, and immediately stored at −80°C.

### 2.3 Metagenome DNA Extraction and Shotgun Sequencing

Total microbial genomic DNA was extracted from fecal samples using the DNeasy PowerSoil Kit (QIAGEN, Netherlands), according to the manufacturer's instructions, and stored at −20°C prior to further analysis. The quantity and quality of the extracted DNA were assessed using a NanoDrop ND‐1000 spectrophotometer (Thermo Fisher Scientific, USA) and agarose gel electrophoresis, respectively. Extracted DNA was used to construct metagenomic shotgun sequencing libraries with an insert size of approximately 400 bp using the Illumina TruSeq Nano DNA LT Library Preparation Kit. Libraries were sequenced on an Illumina HiSeq X‐ten platform (Illumina, USA) using 150‐bp paired‐end (PE150) sequencing at Suzhou PANOMIX Biomedical Tech Co. Ltd.

### 2.4 Metabolite Extraction and Detection

Detailed methods for metabolite extraction and detection from fecal samples are provided in the Supporting Information.

### 2.5 Metagenomics and Metabolomics Analysis

Metagenomic and metabolomic analyses included data preprocessing, statistical analysis, and pathway enrichment analysis. Details are provided in the Supporting Information.

### 2.6 SD Rat Model of CINV

Forty female Sprague–Dawley (SD) rats (6–8 weeks old, body weight 200 ± 20 g) were obtained from SPF Biotechnology Co. Ltd. (Suzhou, China). Gut microbiota transplantation experiments were performed in a specific pathogen‐free (SPF) animal facility. Upon arrival, rats were acclimatized for 1 week. Room temperature was maintained at 22°C ± 2°C with a relative humidity of 55% ± 10%. A 12‐h light/dark cycle was maintained (lights on at 08:00, off at 20:00). Animals had ad libitum access to food and water. Following acclimatization, rats were randomly assigned to four groups (*n* = 10 per group): Control group, Cisplatin model group, non‐CINV microbiota transplantation + cisplatin group (non‐CINV‐cisplatin), and CINV microbiota transplantation + cisplatin group (CINV‐cisplatin). The sample size per group was determined on the basis of similar studies. During the acclimatization period, rats were handled and mock‐gavaged daily with an empty gavage needle to habituate them to the procedures and minimize stress‐related artifacts.

Five days prior to modeling, kaolin pellets were introduced into the feed to allow rats to familiarize themselves with and adapt to their presence. The amounts of kaolin ingested and feed consumed, as well as body weight, were measured and recorded every 24 h. After 3 days of kaolin exposure, rats showing high curiosity towards kaolin while maintaining substantial food intake were identified. Group assignments were reviewed and adjusted if necessary to ensure baseline homogeneity. Modeling and treatment commenced once all rats exhibited minimal interest in kaolin consumption. Two hours before modeling, rats in the non‐CINV‐cisplatin and CINV‐cisplatin groups received a cocktail of non‐absorbable antibiotics (vancomycin [100 mg/kg], neomycin sulfate [200 mg/kg], metronidazole [200 mg/kg], ampicillin [200 mg/kg]) via oral gavage once daily for 5 consecutive days to deplete the resident gut microbiota. Beginning on day 6, fresh fecal samples (5‐10 g) were randomly pooled from 10 CINV patients and 10 non‐CINV patients, respectively. Each pool was homogenized in phosphate‐buffered saline (PBS; 0.125 g/mL), vortexed, and centrifuged at low speed (1000 × g, 1 min) to obtain the supernatant. The supernatant was administered by oral gavage to rats in the corresponding groups (150 μL per rat). After 3 consecutive days of daily administration, the frequency was reduced to every 3 days, and this regimen continued until the experimental endpoint. This procedure was performed without anesthesia.

Cisplatin was administered via a single intraperitoneal injection (6 mg/kg) to the cisplatin model group, non‐CINV‐cisplatin group, and CINV‐cisplatin group. The control group received an equivalent volume of sterile saline via intraperitoneal injection. Seventy‐two hours post‐injection, rats were euthanized under isopentane anesthesia by cervical dislocation, and the medulla oblongata and colon were harvested. These samples were subjected to immunohistochemistry (IHC), RT‐qPCR, and Western blotting. The experiment was performed once, with 3–4 rats per group. No unexpected deaths, severe adverse events, or injection failures occurred during the study. Experimenters were blinded to group assignments until data analysis. Drug injections were randomized across animals, and cage positions were rotated weekly to minimize confounding.

To specifically compare gut microbiota responses to cisplatin in rats transplanted with CINV versus non‐CINV microbiota, resident gut microbiota was depleted by pre‐treatment with a quadruple non‐absorbable antibiotic cocktail (Section 2.7). In accordance with animal ethics principles, modeling of malignant tumors was not required for this microbiota‐focused study.

### 2.7 IHC

IHC was performed per the manufacturer's protocol (detailed in Supporting Informations and Methods). Primary antibodies included rabbit polyclonal anti‐5‐HT3R, anti‐NK1R, and anti‐NK2R. Detailed antibody information was provided in the Supporting Informations and Methods.

Two independent pathologists blinded to experimental groups evaluated protein expression. An immunoreactive score (IRS) system was used to evaluate immunohistochemical results. The percentage of positively stained tumor cells was scored as 0 (< 1%), 1 (1%–10%), 2 (11%–50%), 3 (51%–80%), or 4 (> 80%), and the intensity of staining was scored as 0 (none), 1 (weak), 2 (moderate), or 3 (strong). The final immunoreactive score (IRS) was calculated by multiplying the two scores, yielding a range of 0–12 [21].

### 2.8 RT‐qPCR

Tissue samples were minced and homogenized in liquid nitrogen. Total RNA was extracted using TRIzol regent (Invitrogen) according to the manufacturer's instructions. Reverse transcription was performed using a PrimeScript RT Master Mix Kit (TAKARA). RT‐qPCR was conducted using SYBR Premix Ex Taq (Takara Bio) on a Roche LightCycler 480 system. *GAPDH* was used as the reference gene. Relative mRNA expression was calculated via the 2^(^−ΔΔCt^) method. The primers used were listed in the Supporting Informations and Methods.

### 2.9 Western Blotting

Western blotting was performed according to previously described standard methods. Key steps included: SDS‐PAGE gel preparation, sample loading, electrophoresis, membrane transfer, blocking, incubation with primary and HRP‐conjugated secondary antibodies, and membrane washing. Primary antibodies against 5‐HT3R, NK1R, NK2R, and GAPDH were used; detailed information was provided in the Supporting Informations and Methods. The experiment was performed according to the manufacturer's instructions. GAPDH was used as the internal control. Proteins were detected by enhanced chemiluminescence (ECL) using an iBright FL1500 imaging system. The intensities of the protein bands were determined using ImageJ 1.43 software and compared with those of the GAPDH control.

### 2.10 Statistical Analysis

Metagenomic and metabolomic analyses are described in Supporting Informations. The results of clinical data, in vitro and vivo assays were analyzed using SPSS version 26.0 (IBM Inc., Chicago, IL, USA) and GraphPad Prism 8 (GraphPad Software Inc., San Diego, CA, USA). Data normality was assessed using Shapiro–Wilk tests, and homogeneity of variance was verified via Levene's test. Normally distributed data with homogeneous variance were analyzed by one‐way ANOVA with Tukey's post hoc test. Non‐normally distributed data were analyzed with Kruskal–Wallis tests and Dunn's multiple comparisons. All statistical assumptions and alternative approaches were pre‐specified in the analysis protocol. Statistical significance was defined as *p* < 0.05 (two‐tailed).

## 3 Results

### 3.1 The Characteristics of Included Patients

**FIGURE 1:**
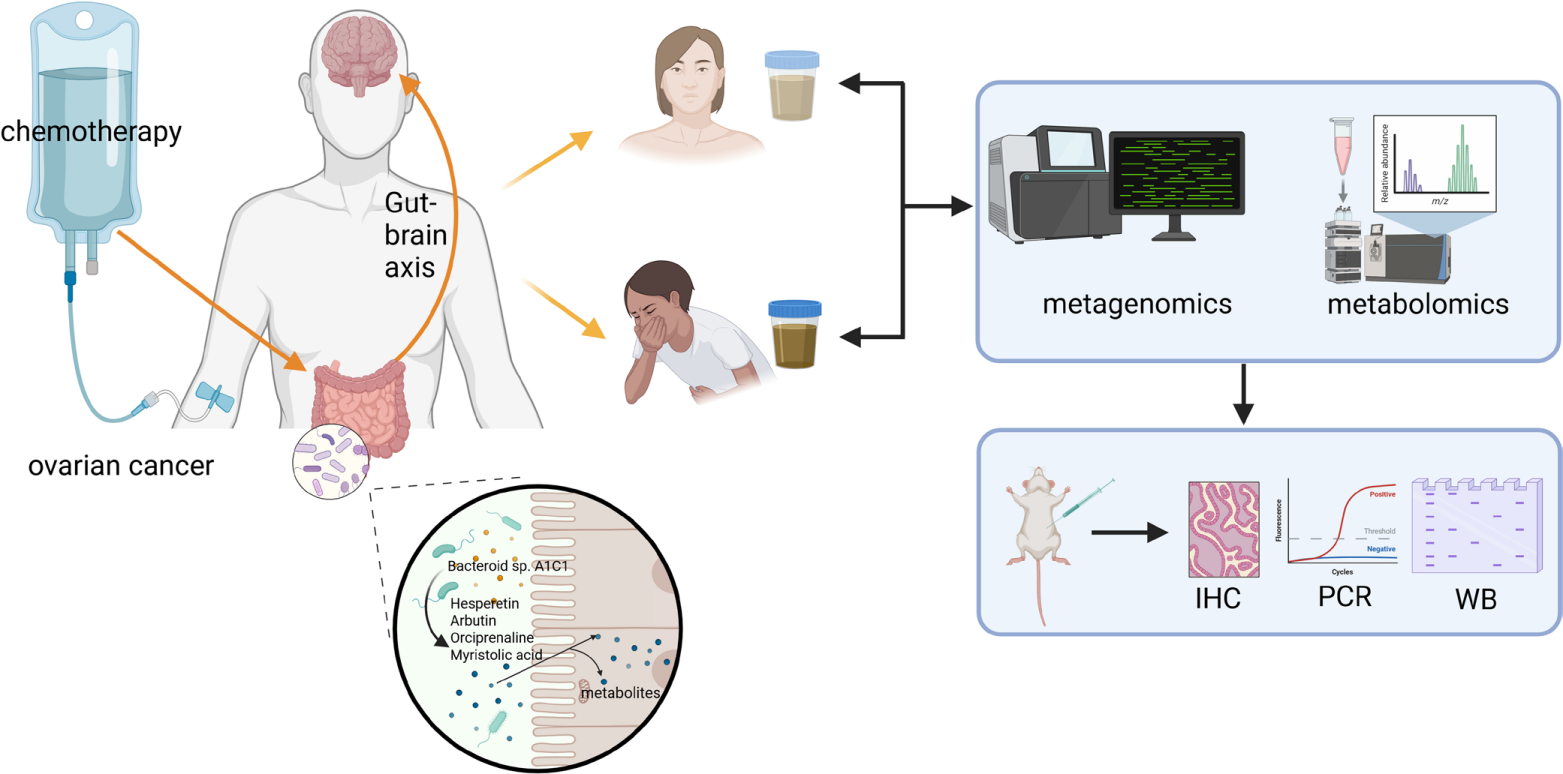
Schematic diagram of the study design. Overview of the integrated clinical and experimental approach: (1) Ovarian cancer patient recruitment and fecal sample collection; (2) metagenomic/metabolomic profiling of CINV vs. non‐CINV groups; (3) fecal microbiota transplantation in SD rats; (4) Cisplatin‐induced CINV modeling and phenotypic assessment.

We performed metagenomic and metabolomic analyses of fecal samples to investigate associations between gut microbiota and CINV development in ovarian cancer patients. Furthermore, we validated the role of gut microbiota in CINV pathogenesis through rat experiments (Figure 1). This integrated approach enhanced the study's rigor, reproducibility, and biological interpretability.

The study enrolled 50 ovarian cancer patients (25 with CINV, 25 without CINV). Their clinical and pathological characteristics are summarized in Table 1.

**TABLE 1:** The clinical and pathological characteristics of 50 patients included in the study.

Characteristic	Total	CINV	Non‐CINV	*p*
Age, mean (SD), years	53.7 ± 9.5	53.3 ± 7.2	54.1 ± 11.4	0.768
Height, mean (SD), cm	156.7 ± 4.9	156.1 ± 4.2	157.3 ± 5.6	0.376
Weight, mean (SD), Kg	56.0 ± 9.3	56.9 ± 9.9	55.1 ± 8.9	0.505
BMI, Kg/m2	22.9 ± 4.1	23.4 ± 4.4	22.3 ± 3.7	0.345
Stage, *n*/%				0.413
I	17 (34)	7 (28)	10 (40)	
II	3 (6)	2 (8)	1 (4)	
III	28 (56)	14 (56)	14 (56)	
IV	2 (4)	2 (8)	0 (0)	
Chemotherapy, *n*/%				0.289
Carboplatin plus paclitaxel	40 (80)	22 (88)	18 (72)	
Cisplatin plus paclitaxel	10 (20)	3 (12)	7 (28)	

Abbreviations: BMI, body mass index; CINV, chemotherapy‐induced nausea and vomiting; SD, standard deviation.

Baseline characteristics did not differ significantly between CINV and non‐CINV groups (Table 1, *p* > 0.05).

### 3.2 Analysis of Gut Microbiota Composition and Function in Fecal Samples

**FIGURE 2:**
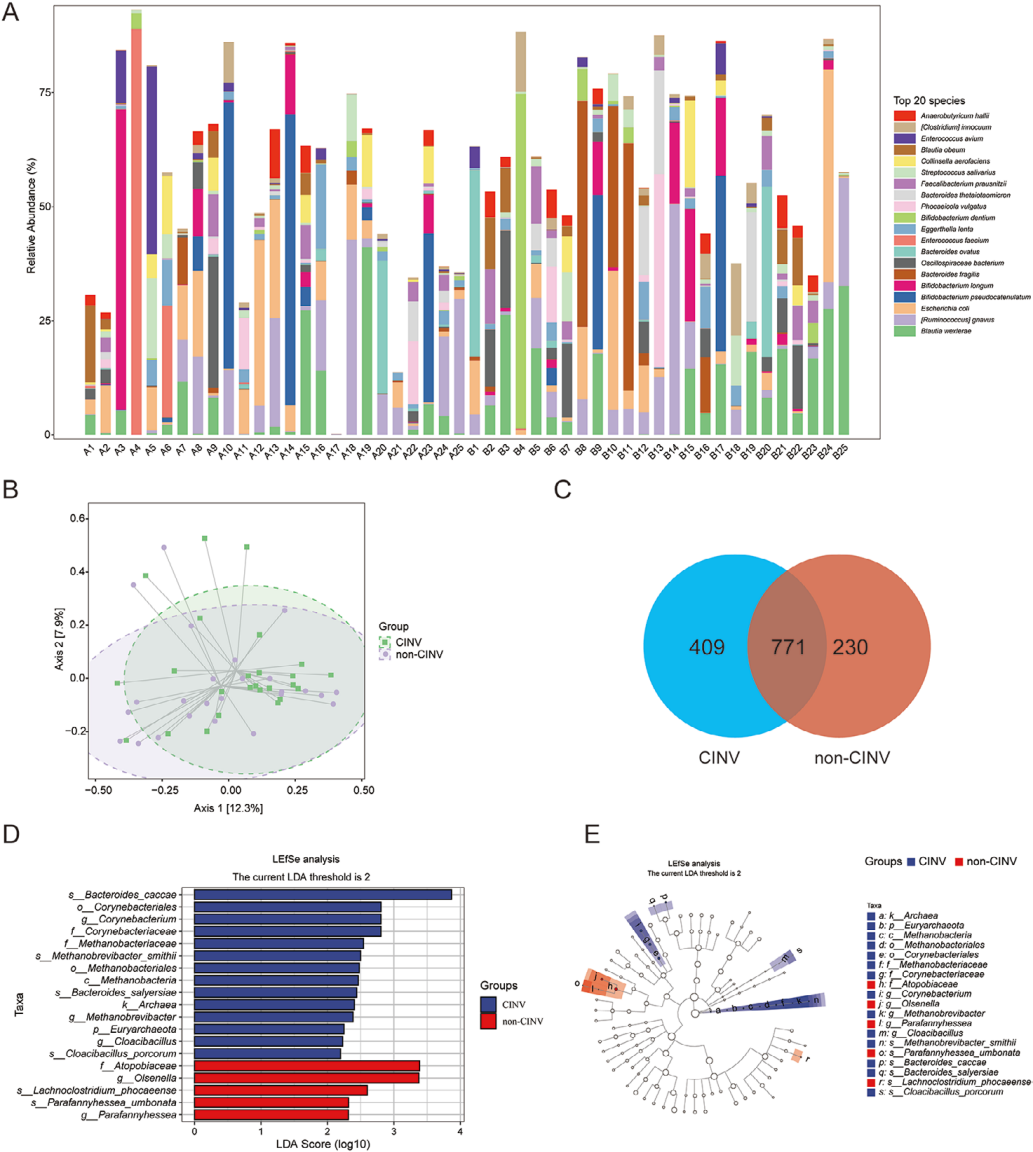
Gut microbiota compositional differences analyzed from human fecal samples. (A) Relative abundance of the top 20 species across samples. (B) Principal component analysis of Beta diversity showing sample clustering patterns. (C) Venn diagram showing species uniqueness/sharing between CINV (blue) and non‐CINV (red) groups. (D) LEfSe analysis identifying discriminant taxa (LDA > 2, *p* < 0.05). Bar length represents LDA score magnitude. (E) Cladogram of phylogenetic distribution of discriminant taxa. Node size reflects relative abundance; colored nodes indicate group‐specific enrichment (blue: CINV; red: Non‐CINV).

Figure 2A displays the top 20 dominant species per sample, including Anaerobutyricum halli, *Clostridium innocuum* , and *Enterococcus avium* . Significant inter‐sample variations in microbial abundance were observed (alpha diversity indices in Table S1). Species accumulation curves indicated adequate sampling depth to capture community diversity (Figure S1A). Species abundance distribution patterns are visualized in Figure S1B.

Principal component analysis of Beta diversity (PCoA; Figure 2B) revealed significant sample clustering patterns. Although samples exhibited partial similarity, NMDS analysis confirmed significant inter‐group dissimilarities (Figure S1C).

Venn analysis identified 409 species unique to CINV patients, 230 unique to non‐CINV, and 771 shared species (Figure 2C). LEfSe analysis revealed group‐specific enrichments (Figure 2D). Among them, the abundance of *Bacteroides caccae* , Corynebacteriales, and Corynebacterium significantly increased in the CINV group, whereas the abundance of Atopobiaceae and Olsenella significantly increased in the non‐CINV group. A cladogram illustrates the phylogenetic distribution of group‐discriminatory taxa (Figure 2E).

**FIGURE 3:**
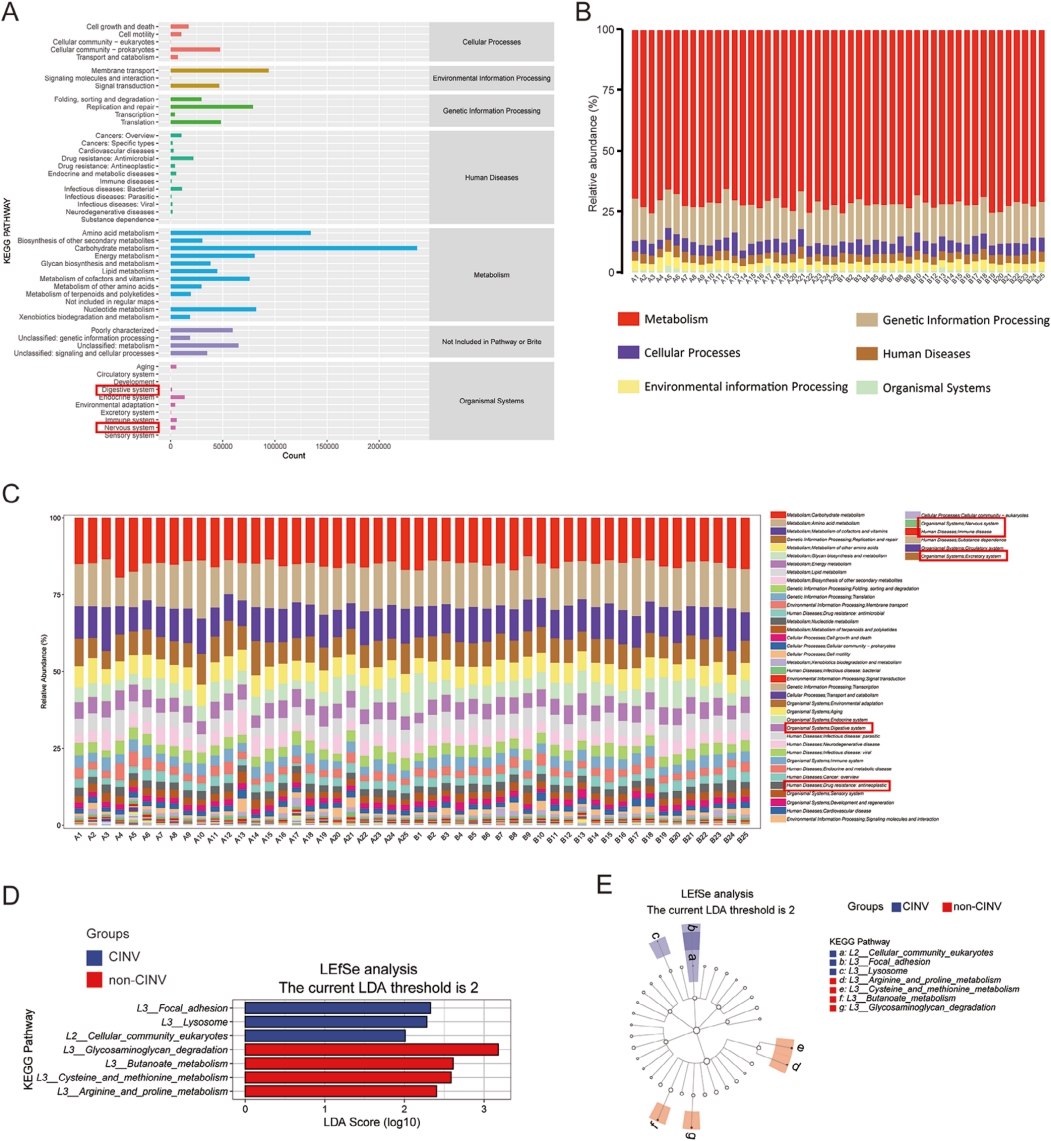
Functional profiling of gut microbiota on the basis of metagenomic data from human fecal samples. (A) KEGG pathway enrichment at level 2. Pathway categories color‐coded by level 1 classification. (B) Relative abundance of level 1 KEGG pathways across samples. (C) Relative abundance of level 2 KEGG pathways. (D) LEfSe analysis of enriched KEGG pathways (LDA > 2, *p* < 0.05). (E) Cladogram of KEGG hierarchy (level 1–3) for discriminant pathways. Node size indicates mean abundance; colored nodes show group‐specific enrichment (blue: CINV; red: Non‐CINV).

The functional analysis of the gut microbiota is shown in Figure 3. The KEGG metabolic pathway enrichment results revealed that the digestive and nervous systems were associated with differential gut microbiota (Figure 3A). Primary and secondary metabolic pathways of KEGG in each sample showed that the digestive system, drug resistance of antineoplastic, nervous system, immune disease, and excretory system were enriched (Figure 3B,C). The results of LEfSe analysis showed significant enrichment of functions in both groups. KEGG pathway enrichment analysis showed that focal adhesion, lysosome, and cellular community eukaryotes were enriched in the CINV group, whereas glycosaminoglycan degradation, butanoate metabolism, cysteine, and methionine metabolism were enriched in the CINV group (Figure 3D). The Cladogram of functional classification displays the hierarchical taxonomic distribution of marker species in each group of samples (Figure 3E).

### 3.3 Metabolomic Profiling of Fecal Samples

**FIGURE 4:**
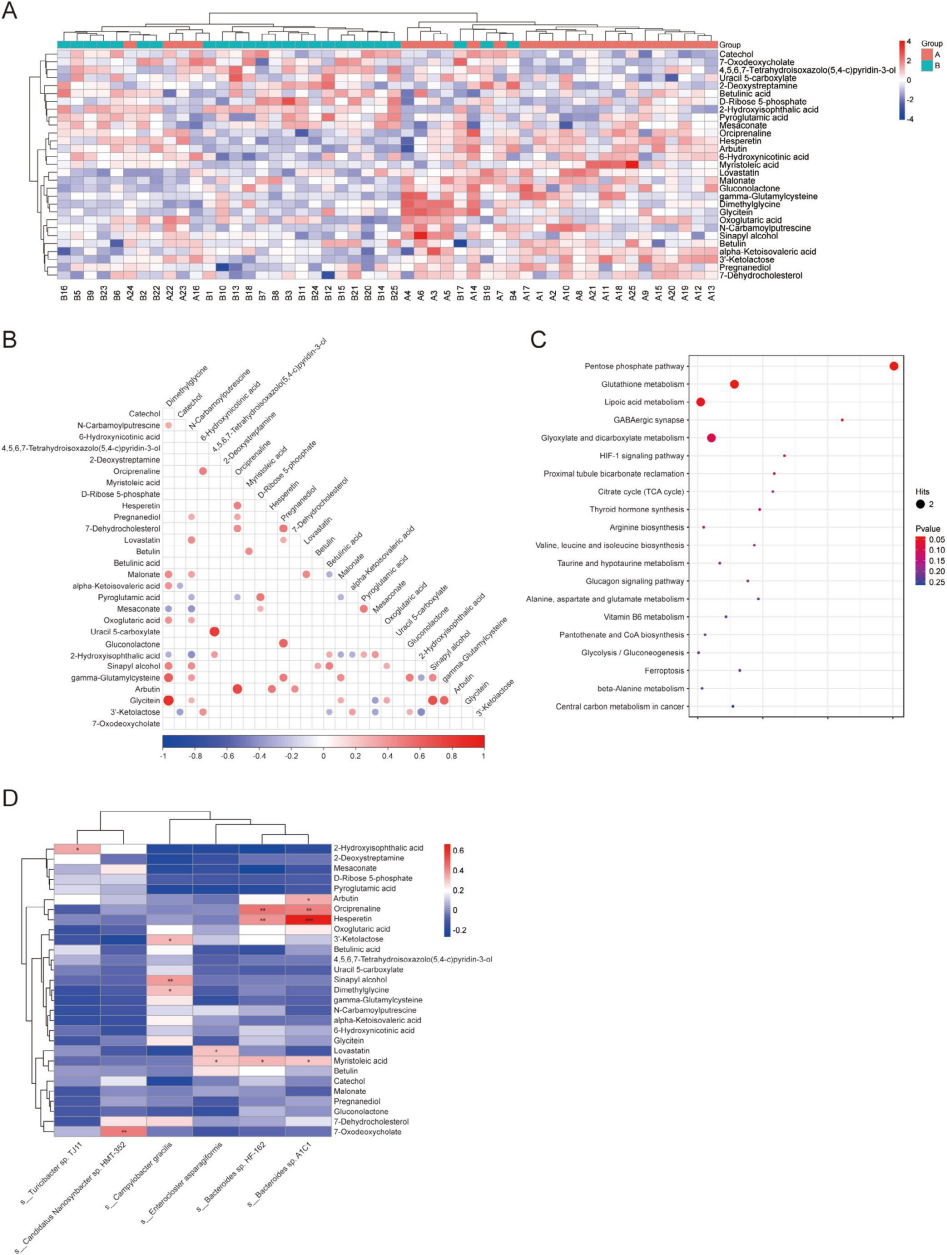
Metabolomic profiling and multi‐omics integration of ovarian cancer fecal samples. (A) Heatmap of 29 differential metabolites from fecal samples (VIP > 1.5, *p* < 0.05). Columns: Metabolites; rows: Samples grouped by CINV status. Red/blue: High/low abundance. (B) Correlation network of significant metabolites (*p* < 0.05). Edge width and color intensity scale with correlation strength. (C) KEGG pathway enrichment of differential metabolites. Dot size reflects metabolite count; dot color indicates *p* value. (D) Bacteria‐metabolite correlation matrix. *p < 0.05, **p < 0.01, ***p < 0.001.

The results of metabolite differential analysis are shown in Figure 4. Level 2 metabolite analysis identified 19 significantly elevated metabolites in CINV patients versus 10 in non‐CINV (Table S2). A heatmap visualizes these differential metabolites (Figure 4A). Correlation analysis revealed strong co‐expression networks among metabolites, particularly hesperetin, arbutin, and orciprenaline (Figure 4B). KEGG enrichment implicated the pentose phosphate pathway, glutathione metabolism, and lipoic acid metabolism in CINV pathogenesis (Figure 4C).

### 3.4 Integrated Analysis of Gut Microbiota and Metabolomics in Fecal Samples

*Bacteroides* sp. A1C1 showed the strongest correlation with hesperetin, arbutin, orciprenaline, and myristolic acid produced. *Bacteroides* sp. HF‐162 was significantly correlated with hesperetin, orciprenaline, and myristolic acid (Figure 4D).

### 3.5 Correlation Analysis Between Gut Microbiota and Severity of Nausea and Vomiting

Nausea severity positively correlated with *Bacteroides* (*r* = 0.691), Parabacteroides (*r* = 0.696), Phocaeicola (*r* = 0.442), Akkermansia (*r* = 0.471), and Ruthenibacterium (*r* = 0.419), but negatively with Enterococcus (*r* = −0.532). Vomiting severity correlated positively with Bacteroides (*r* = 0.571), Parabacteroides (*r* = 0.594), and *Phocaeicola* (*r* = 0.451) (Table S3).

### 3.6 Correlation Analysis Between Microbial Metabolites and Severity of Nausea and Vomiting

Nausea severity positively correlated with 6‐hydroxynicotinate, orciprenaline, myristolic acid, hesperetin, 7‐dehydrocholesterol, betulin, α‐ketoisovalerate, oxoglutarate, arbutin, and 3‐ketolactose (*r* = 0.304–0.748, *p* < 0.05), but negatively with catechol, 2‐deoxystreptamine, mesaconate, uracil‐5‐carboxylate, 2‐hydroxyisophthalate, and 7‐oxodeoxycholate (*r* = −0.296 to −0.386; *p* < 0.05). Vomiting severity showed analogous correlations with these metabolites (Table S4). Notably, these nausea‐ and vomiting‐associated metabolites were themselves significantly correlated with the abundance of specific gut microbiota, particularly Bacteroides sp. A1C1 and HF‐162, as detailed in the integrated multi‐omics analysis (Section 3.4 and Figure 4D).

### 3.7 SD Rat Model of CINV

#### 3.7.1 General Situation

**FIGURE 5:**
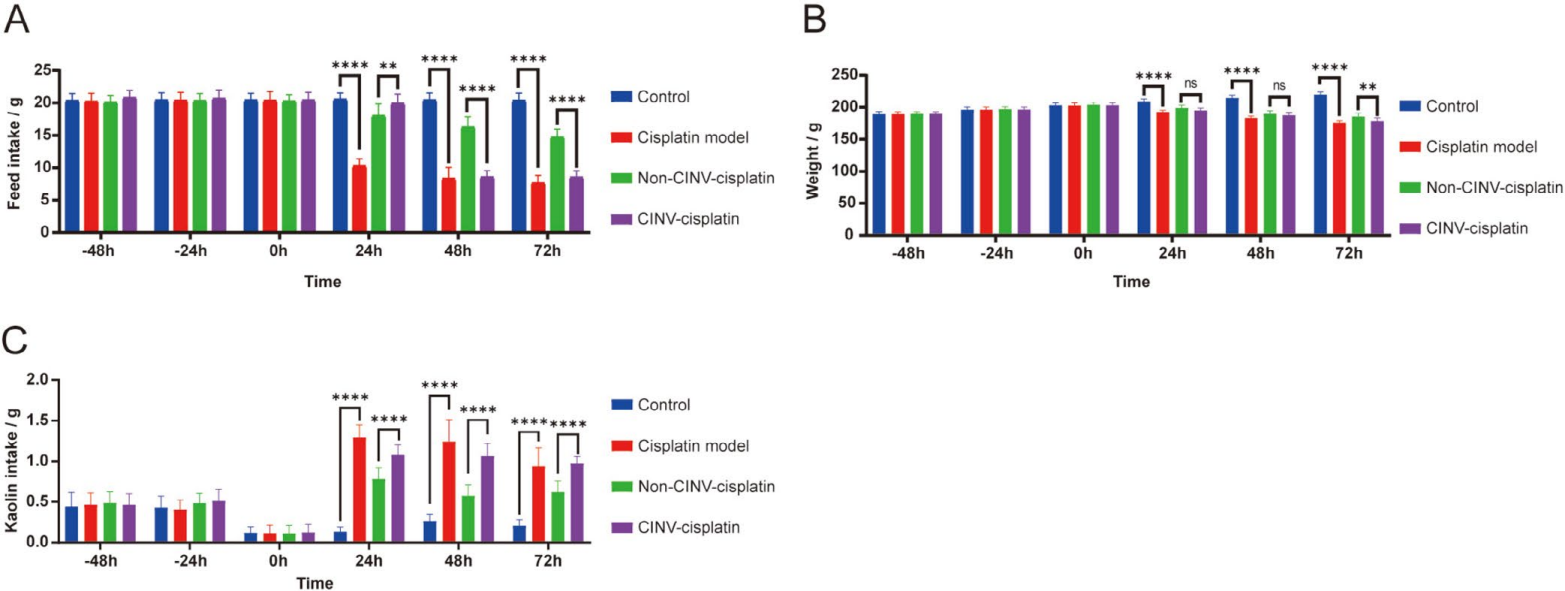
Behavioral and physiological metrics measured in rats. (A) Feed intake (g/24 h). (B) Body weight change (g/24 h). (C) Kaolin consumption (g/24 h). Data are presented as mean ± SD; *n* = 10 rats per group. *p < 0.05, **p < 0.01, ***p < 0.001, ****p < 0.0001.

Prior to modeling, all rats in the four groups (*n* = 10 per group) exhibited normal behavior, including good mental status, alertness, smooth fur, normal food and water intake, urination, and defecation. Within 24 to 72 h post‐cisplatin administration, rats exhibited signs of discomfort, including reduced activity, hunching, lethargy, delayed responses, and ruffled fur. Feed intake and body weight at 24, 48, and 72 h were significantly lower in the cisplatin model group compared to the normal control group (*p* < 0.05), confirming the successful establishment of the CINV rat model. At 24, 48, and 72 h post‐modeling, rats in the non‐CINV‐ cisplatin group showed improvement in clinical signs compared to the CINV‐ cisplatin group. Their feed intake and body weight were significantly higher (*p* < 0.05), indicating amelioration of cisplatin‐induced discomfort. Rats in the normal control group maintained normal mental status, activity levels, diet, and body weight throughout the experiment. Changes in feed intake and body weight across the four groups before and after modeling are presented in Tables S5, S6 and Figure 5A,B. Cisplatin model, non‐CINV microbiota transplantation cisplatin, and CINV microbiota transplantation cisplatin groups.

#### 3.7.2 Kaolin Intake of Pica

Kaolin intake was significantly higher in the cisplatin model group compared to the normal control group at 24, 48, and 72 h post‐modeling (*p* < 0.05), validating the chemotherapy‐induced pica (nausea/vomiting surrogate) rat model. Compared to the CINV‐cisplatin group, rats in the non‐CINV‐cisplatin group exhibited significantly lower kaolin intake at 24, 48, and 72 h (*p* < 0.05), demonstrating a significant amelioration of chemotherapy‐induced pica. Kaolin intake for all four groups before and after modeling is detailed in Table S7 and Figure 5C.

#### 3.7.3 Expression of 5‐HT3R, NK1R, and NK2R in the Rat Medulla Oblongata

**FIGURE 6:**
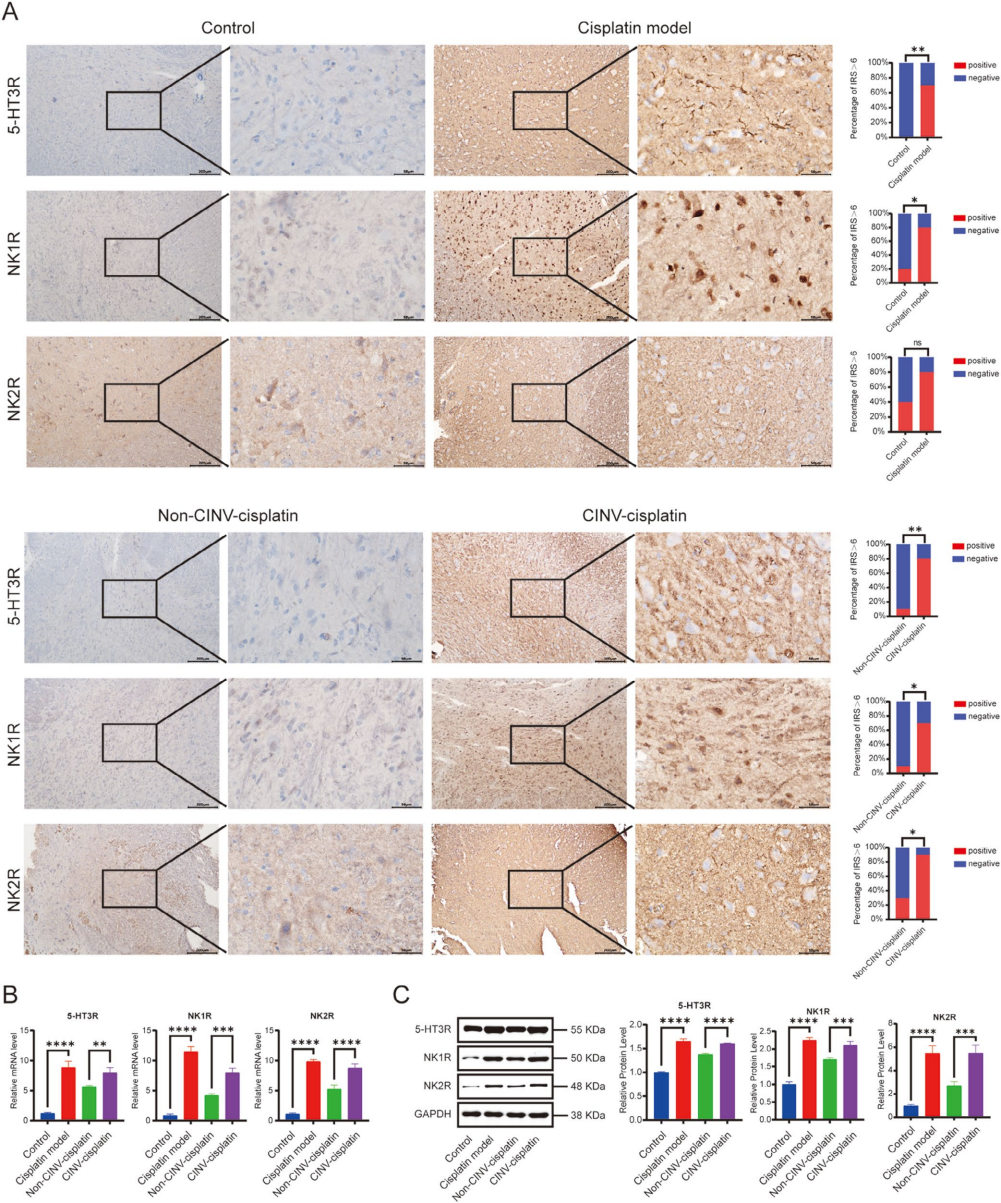
Expression of emesis‐related receptors in the rat medulla oblongata. (A) Representative IHC images (left; scale bar: 200 μm) and quantification of immunoreactive scores (IRS; right). (B) mRNA levels by RT‐qPCR (5‐HT3R, NK1R, and NK2R). (C) Western blot bands (left) and densitometric analysis (right) of protein expression normalized to GAPDH. Tissue samples were collected from the medulla oblongata of rats. *p < 0.05, **p < 0.01, ***p < 0.001, ****p < 0.0001.

The medulla oblongata controls the reaction process of nausea and vomiting, and the related phenotypic proteins of nausea and vomiting include 5‐HT3R, NK1R, and NK2R. Immunohistochemistry revealed cytoplasmic localization of 5‐HT3R and NK2R, and nuclear/cytoplasmic expression of NK1R in medullary neurons. Versus the control group, the cisplatin model group exhibited significantly upregulated medullary 5‐HT3R, NK1R, and NK2R protein expression (*p* < 0.05). Moreover, versus the CINV‐cisplatin group, the non‐CINV‐cisplatin group showed a significant reduction in these proteins (*p* < 0.05) (Figure 6A).

The results of RT‐qPCR and Western Blotting showed that compared with the control group, the mRNA and protein expression of 5‐HT3R, NK1R, and NK2R genes were significantly increased in the medulla oblongata of the cisplatin model group rats (*p* < 0.05). Furthermore, compared with the CINV‐cisplatin group, the mRNA and protein expression of 5‐HT3R, NK1R, and NK2R genes were significantly reduced in the medulla oblongata in the non‐CINV‐cisplatin group (*p* < 0.05) (Figure 6B,C).

#### 3.7.4 Expression of 5‐HT3R, NK1R, and NK2R in the Colon of Model Rats

**FIGURE 7:**
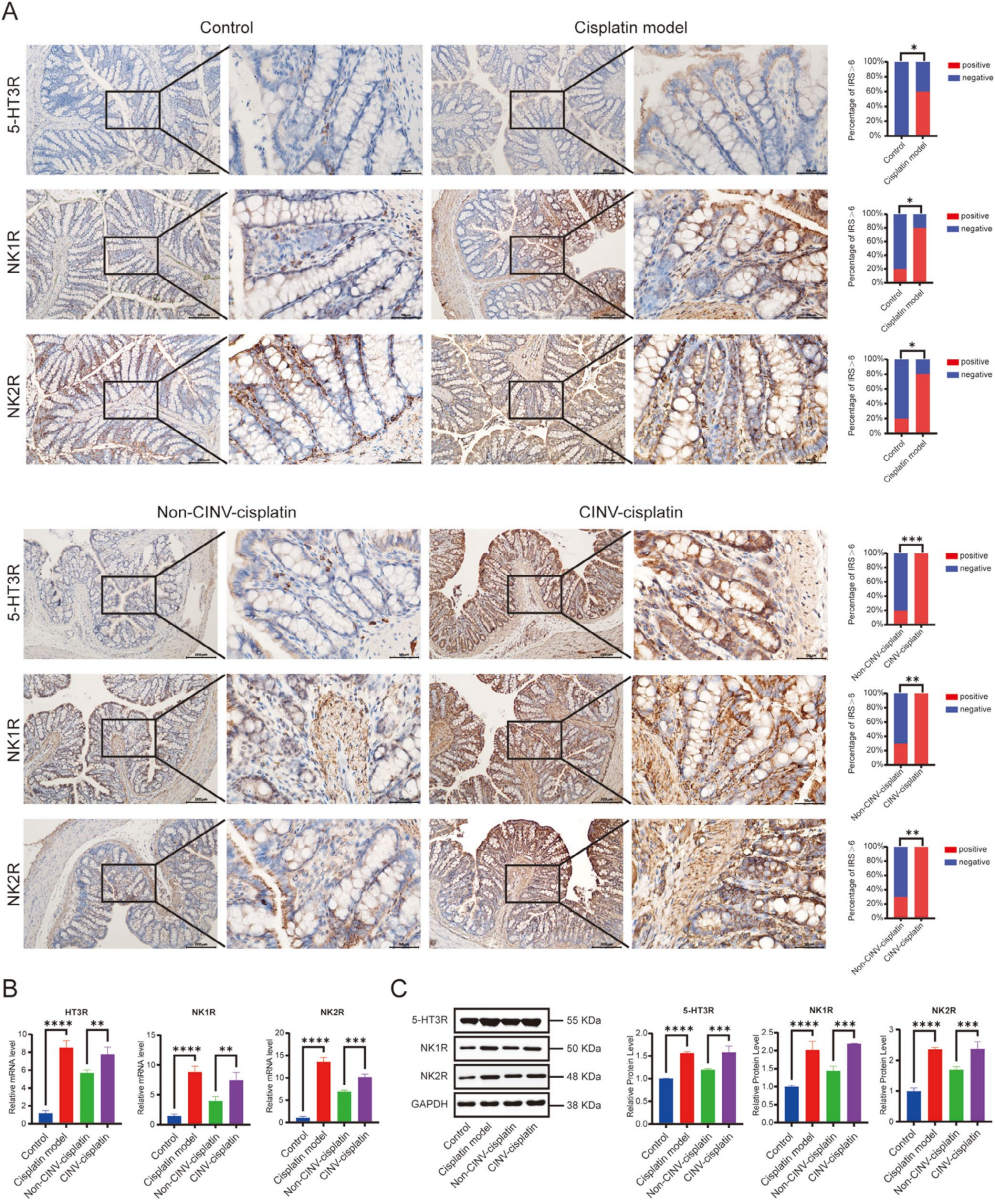
Expression of emesis‐related receptors in the rat colon. (A) Representative IHC images (left; scale bar: 200 μm) and quantification of immunoreactive scores (IRS; right). (B) mRNA levels by RT‐qPCR (5‐HT3R, NK1R, and NK2R). (C) Western blot bands (left) and densitometric analysis (right) of protein expression normalized to GAPDH. Tissue samples were collected from the colon of rats. *p < 0.05, **p < 0.01, ***p < 0.001, ****p < 0.0001.

Similar to medullary findings, colonic 5‐HT3R and NK2R localized to the cytoplasm, whereas NK1R showed nuclear/cytoplasmic expression in absorptive epithelial cells. Compared to the control group, the mRNA and protein expression of 5‐HT3R, NK1R, and NK2R genes were significantly increased in the colon of the cisplatin model group rats (*p* < 0.05). Furthermore, compared with the CINV‐cisplatin group, the mRNA and protein expression of 5‐HT3R, NK1R, and NK2R genes were significantly reduced in the colon in the non‐CINV‐cisplatin group (*p* < 0.05) (Figure 7A).

The results of RT‐qPCR and Western Blotting experiments showed that compared with the normal control group, the colon 5‐HT3R, NK1R, and NK2R gene transcription and protein expression in the cisplatin model group were significantly increased (*p* < 0.05). In addition, compared with the CINV microbiota transplantation cisplatin group, the colon 5‐HT3R, NK1R, and NK2R gene transcription and protein expression in the non‐CINV microbiota transplantation cisplatin group were significantly reduced (*p* < 0.05) (Figure 7B,C).

## 4 Discussion

This study employed metagenomic and metabolomic multi‐omics approaches to investigate the relationship between the gut microbiota and the development of CINV. Our findings demonstrate that pre‐existing differences in the gut microbiota and metabolome at baseline are associated with the subsequent development of CINV. In addition, *Bacteroides* sp. A1C1 and its putatively identified metabolites—hesperetin, arbutin, orciprenaline, and myristolic acid—represent potential diagnostic biomarkers for CINV. Experiments in SD rats further confirmed that the gut microbiota is involved in CINV pathogenesis. Furthermore, modulation of the gut microbiota composition can ameliorate CINV symptoms.

Chemotherapy is an important component and common treatment method for malignant tumors [22]. CINV is a common side effect of chemotherapy in patients with ovarian cancer, which seriously affects their compliance with chemotherapy and treatment efficacy. Previous studies have explained the relationship between gut microbiota and the onset of CINV from the perspective of the gut microbiota [23, 24, 25]. The composition of the gut microbiota is easily influenced by various factors, such as age, disease, diet, and medication use. Therefore, this study focused on exploring the relationship between gut microbiota and CINV before chemotherapy with carboplatin or cisplatin for ovarian cancer.

Previous studies indicate that chemotherapy alters microbial community structure and can significantly reduce the abundance and diversity of the gastrointestinal microbiota. Additionally, chemotherapy often leads to a significant shift from Gram‐positive to Gram‐negative bacteria, characterized by an overall increase in the abundance of *Bacteroidetes* and *Proteobacteria* phyla, and a decrease in *Firmicutes* and *Actinobacteria* [26, 27]. These studies also reported increased abundance of Gram‐negative bacteria, including *Bacteroides caccae* and *Bacteroides salyersiae* (both within the genus *Bacteroides*), in patients experiencing CINV [26, 27]. This suggests that *Bacteroidetes* may become dominant post‐chemotherapy, potentially contributing to CINV development.

Gut microbiota alterations exhibit significant disease‐ and treatment‐specific variations, as demonstrated in acute myeloid leukemia studies examining chemotherapy effects. Consistent with previous reports, we observed a progressive reduction in overall microbial diversity post‐chemotherapy. Specifically, the obligate anaerobe *Blautia* decreased in abundance, whereas lactobacilli increased. However, compared to healthy controls, relative abundances of Lactobacillus, Bacteroides, Bifidobacterium, and Enterococcus were significantly reduced [28, 29, 30]. In our cohort, CINV patients showed significantly increased abundance of *Bacteroides caccae* , Corynebacteriales (order), and *Corynebacterium*, whereas non‐CINV patients exhibited elevated Atopobiaceae (family) and *Olsenella*. Thus, although intestinal dysbiosis represents a universal consequence of cancer therapies, the specific compositional shifts vary substantially across cancer types and treatment regimens.

Our findings regarding specific microbial shifts, particularly the enrichment of *Bacteroides caccae* , invite a comparison with microbiota alterations observed in other cancer types treated with chemotherapy. A degree of consistency exists; for instance, studies in colorectal cancer patients receiving platinum‐based regimens have also reported an increased abundance of bacteria from the *Bacteroidetes* phylum post‐chemotherapy [31]. This suggests that the expansion of *Bacteroides* may represent a common gut microbial response to chemotherapy‐induced stress, potentially contributing to CINV pathogenesis across different cancers. However, the specific enrichment of Corynebacteriales and *Corynebacterium* observed in our cohort has not been as prominently featured in reports from colorectal cancer or breast cancer [31, 32]. These discrepancies likely underscore the influence of disease‐specific factors. The unique pathophysiology of ovarian cancer, including its frequent peritoneal dissemination and the associated inflammatory microenvironment, may create a selective pressure that shapes a distinct gut microbiota profile. Furthermore, variations in chemotherapy dosage, adjunct medications, and baseline patient characteristics could all contribute to these differences. Thus, although certain aspects of gut dysbiosis may be generalized across cancer therapies, the specific compositional shifts are likely modulated by a complex interplay of treatment and disease‐related factors.

Building on these findings, we specifically investigated CINV pathogenesis in ovarian cancer. Integrated metagenomic and metabolomic analyses revealed significant correlations between gut microbial communities/metabolites and CINV status. Strain‐level correlation analysis identified *Bacteroides* sp. A1C1 and *Bacteroides* sp. HF‐162 is strongly associated with CINV. These strains showed significant co‐occurrence with the metabolites hesperetin, arbutin, orciprenaline, and myristolic acid, suggesting their involvement in CINV pathogenesis. Although gut dysbiosis has been linked to ovarian cancer progression and platinum resistance, its specific role in CINV pathogenesis remains unexplored [33, 34, 35]. Our study, therefore, provides novel insights into the gut microbiota‐CINV relationship in ovarian cancer. Future studies should validate the causative roles of *Bacteroides* sp. A1C1 and HF‐162 in CINV pathogenesis.

Among the top 20 differentially abundant taxa, *Bacteroides*, *Parabacteroides*, and *Phocaeicola* showed significant correlations with nausea and vomiting severity scores. Analysis of the 29 differential metabolites revealed strong positive correlations between nausea severity and levels of arbutin (*r* = 0.399), orciprenaline (*r* = 0.411), hesperetin (*r* = 0.386), and myristoleic acid (*r* = 0.328; all *p* < 0.05). Similarly, vomiting severity positively correlated with these metabolites (arbutin *r* = 0.334, orciprenaline *r* = 0.324, hesperetin *r* = 0.322, myristolic acid *r* = 0.292; all *p* < 0.05). These microbial and metabolic signatures represent promising diagnostic biomarkers for CINV.

Through integrated multi‐omics approaches, we established significant associations between gut microbiota profiles and CINV in ovarian cancer patients. To verify causality, we conducted fecal microbiota transplantation (FMT) studies in rats. Recipients of non‐CINV microbiota exhibited significantly reduced intake of pica kaolin, and the expression of nausea and vomiting‐related phenotype proteins 5‐HT3R, NK1R, and NK2R in the medulla oblongata and colon was also significantly reduced. These findings demonstrate that gut microbiota directly contributes to CINV pathogenesis, and that microbiota modulation can ameliorate CINV symptoms. It provides compelling evidence for the gut‐brain axis's role in CINV [36, 37]. This systemic effect raises the question of the communication route. It is plausible that microbiota‐derived metabolites enter the systemic circulation to directly influence the central nervous system [36, 37]. An equally compelling mechanism involves the modulation of gut‐based receptor expression and subsequent signaling to the brainstem via vagal afferents [15, 36]. These pathways are not mutually exclusive and may operate in concert. Future research that directly quantifies identified microbial metabolites in the serum and cerebrospinal fluid of model animals will be crucial to distinguish the relative contributions of the humoral and neural pathways.

Although our FMT study in healthy rats provides direct evidence for the causal role of gut microbiota in CINV, we acknowledge that this model does not fully recapitulate the complex pathophysiology of a cancer‐bearing host. The absence of an ovarian tumor burden and associated systemic inflammation is a limitation of our current experimental design. It is plausible that the presence of a tumor and its microenvironment could modulate the gut‐brain axis and potentially alter the efficacy of microbiota‐based interventions. Therefore, our findings establish a foundational principle that gut microbiota composition directly influences CINV susceptibility [37, 38]. Future studies employing orthotopic ovarian cancer models will be invaluable to validate and extend these findings within the context of cancer‐specific physiology and to explore potential interactions between the tumor microenvironment and the gut microbiota in driving CINV.

This study has several limitations. First, the sample size (*n* = 50) from a single center may limit the generalizability of our findings and leaves the potential for unmeasured confounding factors, such as detailed dietary habits and psychological stress, despite our efforts to control for major clinical variables and medication use. It is noteworthy, however, that comparable sample sizes have been employed in seminal gut microbiome studies utilizing deep multi‐omics profiling for hypothesis generation [39, 40]. Therefore, although our study provides robust initial evidence, future larger‐scale, multi‐center cohorts are essential to confirm and extend our findings. Second, although multi‐omics analysis implicated specific bacterial strains such as *Bacteroides* sp. A1C1 and *Bacteroides* sp. HF‐162 in CINV pathogenesis, their causative roles remain unconfirmed due to the unavailability of these strains for functional validation. Consequently, the potential biomarkers identified here require validation in larger, multi‐center cohorts, and future studies utilizing isolated strains are needed to confirm their pathogenic roles and therapeutic mechanisms in CINV.

In conclusion, pre‐existing differences in the gut microbiota and metabolome at baseline are significantly associated with the incidence and severity of CINV in ovarian cancer patients. Specifically, the gut microbiota species *Bacteroides* sp. A1C1 and *Bacteroides* sp. HF‐162, along with its putatively identified metabolites (hesperetin, arbutin, orciprenaline, and myristoleic acid), represent potential diagnostic biomarkers for CINV. Furthermore, the gut microbiota plays a role in CINV pathogenesis, and modulating its composition can ameliorate CINV symptoms.

## Author Contributions

**Shuiling Zu:** methodology, writing – original draft, and funding acquisition. **Xiaoyan Yu:** methodology, writing – original draft, and formal analysis. **Jihong Song:** validation, resources, and data curation. **Yu Xiao:** investigation, and visualization. **Huan Yi:** conceptualization, writing – review and editing, and supervision. **Hong Li:** conceptualization, writing – review and editing, and project administration. All authors have read and approved the final manuscript.

## Funding

This work was supported by Startup Fund for Scientific Research, Fujian Medical University (2023QH1193) and Nursing Research Special Fund of Fujian Maternal and Child Health Hospital (YCXH 22–10).

## Ethics Statement

This study was approved by the Institutional Review Board of the Fujian Maternity and Child Health Hospital (2023KY188). Animal experiments were approved by the Institutional Animal Care and Use Committee (IACUC) of Fujian Maternity and Child Health Hospital (AEC‐SFY‐2025‐012). All patients signed an informed consent form. This study was conducted in accordance with the principles of the Declaration of Helsinki. Patient identities were not identified in the study.

## Conflicts of Interest

The authors declare no conflicts of interest.

## Supporting information


**Data S1:** Supporting Information.

**Figure S1:** Microbial diversity analyses. (A) Species accumulation curve. X‐axis: Number of samples sequenced; Y‐axis: Observed species richness. The shaded area indicates 95% confidence interval. Curve plateau demonstrates adequate sampling depth. (B) Rank‐abundance distribution. Species ranked by relative abundance (high to low). Y‐axis: log2 transformed relative abundance. (C) Non‐metric multidimensional scaling (NMDS; stress = 0.15). Ordination plot showing sample clustering by group. Stress value < 0.2 indicates good ordination representation.
**Table S1:** Results of calculating the alpha diversity of different sample species using different metric indices.
**Table S2:** Statistical results from the fecal metabolomics data comparison between the CINV group and the non‐CINV group.
**Table S3:**. Correlation analysis between gut microbial genera and the severity of nausea and vomiting symptoms.
**Table S4:**. Correlation analysis between differential metabolites and the severity of nausea and vomiting symptoms.
**Table S5:**. Changes in the feed intake of rats before and after modeling.
**Table S6:**. Changes in body weight of rats before and after modeling.
**Table S7:**. Changes in kaolin intake of rats before and after modeling.

## Data Availability

The raw metagenomic sequencing data have been deposited in the NCBI Sequence Read Archive under BioProject accession number PRJNA1290541 (https://www.ncbi.nlm.nih.gov/). The raw metabolomics data have been deposited in the China National Center for Bioinformation (CNCB) under accession number PRJCA042692 (https://ngdc.cncb.ac.cn/omix/preview/vK8M2F4C). The other data that support the findings of this study are available from the corresponding authors, Huan Yi and Hong Li, upon reasonable request.

## References

[1] H. Sung , J. Ferlay , R. L. Siegel , et al., “Global Cancer Statistics 2020: GLOBOCAN Estimates of Incidence and Mortality Worldwide for 36 Cancers in 185 Countries,” CA: A Cancer Journal for Clinicians 71 (2021): 209–249. 33538338 10.3322/caac.21660

[2] P. Patel , E. Paw Cho Sing , and L. L. Dupuis , “Safety of Clinical Practice Guideline‐Recommended Antiemetic Agents for the Prevention of Acute Chemotherapy‐Induced Nausea and Vomiting in Pediatric Patients: A Systematic Review and Meta‐Analysis,” Expert Opinion on Drug Safety 18 (2019): 97–110. 30640557 10.1080/14740338.2019.1568988

[3] K. Gupta , R. Walton , and S. P. Kataria , “Chemotherapy‐Induced Nausea and Vomiting: Pathogenesis, Recommendations, and New Trends,” Cancer Treatment and Research Communications 26 (2021): 100278. 33360668 10.1016/j.ctarc.2020.100278

[4] D. Gala , H. H. Wright , B. Zigori , S. Marshall , and M. Crichton , “Dietary Strategies for Chemotherapy‐Induced Nausea and Vomiting: A Systematic Review,” Clinical Nutrition 41 (2022): 2147–2155. 36067586 10.1016/j.clnu.2022.08.003

[5] Y. He , J. Zheng , B. Ye , Y. Dai , and K. Nie , “Chemotherapy‐Induced Gastrointestinal Toxicity: Pathogenesis and Current Management,” Biochemical Pharmacology 216 (2023): 115787. 37666434 10.1016/j.bcp.2023.115787

[6] G. James and B. Alberto , “Complementary Pharmacokinetic Profiles of Netupitant and Palonosetron Support the Rationale for Their Oral Fixed Combination for the Prevention of Chemotherapy‐Induced Nausea and Vomiting,” Journal of Clinical Pharmacology 59, no. 4 (2019): 472–487. 30412271 10.1002/jcph.1338 PMC6587462

[7] G. Atefeh , J. Hossein , R. A. Amir , and H. Seyed Isaac , “Investigation of the Role of Neurokinin‐1 Receptor Inhibition Using Aprepitant in the Apoptotic Cell Death Through PI3K/Akt/NF‐κB Signal Transduction Pathways in Colon Cancer Cells,” BioMed Research International 2021 (2021): 1383878. 34395609 10.1155/2021/1383878 PMC8355960

[8] C. Yi , W. Zehua , S. Lishuo , et al., “Aprepitant Plus Palonosetron Versus Dexamethasone Plus Palonosetron in Preventing Chemotherapy‐Induced Nausea and Vomiting in Patients With Moderate‐Emetogenic Chemotherapy: A Randomized, Open‐Label, Phase 3 Trial,” EClinicalMedicine 49 (2022): 101480. 35747189 10.1016/j.eclinm.2022.101480 PMC9167865

[9] S. P. Levick , D. R. Soto‐Pantoja , J. Bi , et al., “Doxorubicin‐Induced Myocardial Fibrosis Involves the Neurokinin‐1 Receptor and Direct Effects on Cardiac Fibroblasts,” Heart, Lung & Circulation 28, no. 10 (2019): 1598–1605. 10.1016/j.hlc.2018.08.003 PMC7901001 30205930

[10] R. M. Navari , “The Safety of Antiemetic Medications for the Prevention of Chemotherapy‐Induced Nausea and Vomiting,” Expert Opinion on Drug Safety 15 (2016): 343–356. 26699406 10.1517/14740338.2016.1135899

[11] S. Sommariva , B. Pongiglione , and R. Tarricone , “Impact of Chemotherapy‐Induced Nausea and Vomiting on Health‐Related Quality of Life and Resource Utilization: A Systematic Review,” Critical Reviews in Oncology/Hematology 99 (2016): 13–36. 26697988 10.1016/j.critrevonc.2015.12.001

[12] H. Marie , H. Arash , I. Claudia , et al., “The 5‐HT(3) Receptor Affects Rotavirus‐Induced Motility,” Journal of Virology 95, no. 15 (2021): e0075121. 33980599 10.1128/JVI.00751-21 PMC8274622

[13] M. C. Janelsins , M. A. Tejani , C. Kamen , A. R. Peoples , K. M. Mustian , and G. R. Morrow , “Current Pharmacotherapy for Chemotherapy‐Induced Nausea and Vomiting in Cancer Patients,” Expert Opinion on Pharmacotherapy 14 (2013): 757–766. 23496347 10.1517/14656566.2013.776541 PMC3938333

[14] P. Singh , S. S. Yoon , and B. Kuo , “Nausea: A Review of Pathophysiology and Therapeutics,” Therapeutic Advances in Gastroenterology 9 (2016): 98–112. 26770271 10.1177/1756283X15618131 PMC4699282

[15] B. L. Rapoport , “Delayed Chemotherapy‐Induced Nausea and Vomiting: Pathogenesis, Incidence, and Current Management,” Frontiers in Pharmacology 8 (2017): 19. 28194109 10.3389/fphar.2017.00019 PMC5277198

[16] B. C. Song and J. Bai , “Microbiome‐Gut‐Brain Axis in Cancer Treatment‐Related Psychoneurological Toxicities and Symptoms: A Systematic Review,” Supportive Care in Cancer 29 (2021): 605–617. 32918608 10.1007/s00520-020-05739-9 PMC7769970

[17] J. E. Bajic , I. N. Johnston , G. S. Howarth , and M. R. Hutchinson , “From the Bottom‐Up: Chemotherapy and Gut‐Brain Axis Dysregulation,” Frontiers in Behavioral Neuroscience 12 (2018): 104. 29872383 10.3389/fnbeh.2018.00104 PMC5972222

[18] K. R. Jordan , B. R. Loman , M. T. Bailey , and L. M. Pyter , “Gut Microbiota‐Immune‐Brain Interactions in Chemotherapy‐Associated Behavioral Comorbidities,” Cancer 124 (2018): 3990–3999. 29975400 10.1002/cncr.31584 PMC6234095

[19] S. Zhong , Z. Zhou , Y. Liang , et al., “Targeting Strategies for Chemotherapy‐Induced Peripheral Neuropathy: Does Gut Microbiota Play a Role?,” Critical Reviews in Microbiology 45 (2019): 369–393. 31106639 10.1080/1040841X.2019.1608905

[20] National Cancer Institute Division of Cancer Treatment and Diagnosis , “Common Terminology Criteria for Adverse Events (CTCAE),” https://ctep.cancer.gov/protocolDevelopment/electronic_applications/ctc.htm#ctc_40.

[21] E. Zhang , Y. Zhang , Z. Fan , L. Cheng , S. Han , and H. Che , “Apigenin Inhibits Histamine‐Induced Cervical Cancer Tumor Growth by Regulating Estrogen Receptor Expression,” Molecules 25, no. 8 (2020): 1960. 32340124 10.3390/molecules25081960 PMC7221565

[22] D. H. Suh , M. Kim , K. H. Lee , et al., “Major Clinical Research Advances in Gynecologic Cancer in 2017,” Journal of Gynecologic Oncology 29 (2018): e31. 29468855 10.3802/jgo.2018.29.e31 PMC5823987

[23] K. Singh , H. Cao , C. Miaskowski , et al., “Perturbations in Endocytotic and Apoptotic Pathways Are Associated With Chemotherapy‐Induced Nausea,” Biological Research for Nursing 23 (2021): 238–247. 32815385 10.1177/1099800420951271 PMC8822189

[24] J. Reyna‐Figueroa , E. Barron‐Calvillo , C. Garcia‐Parra , et al., “Probiotic Supplementation Decreases Chemotherapy‐Induced Gastrointestinal Side Effects in Patients With Acute Leukemia,” Journal of Pediatric Hematology/Oncology 41 (2019): 468–472. 31033786 10.1097/MPH.0000000000001497

[25] S. M. Ervin , S. V. Ramanan , and A. P. Bhatt , “Relationship Between the Gut Microbiome and Systemic Chemotherapy,” Digestive Diseases and Sciences 65 (2020): 874–884. 32026181 10.1007/s10620-020-06119-3 PMC7046092

[26] E. Montassier , E. Batard , S. Massart , et al., “16S rRNA Gene Pyrosequencing Reveals Shift in Patient Faecal Microbiota During High‐Dose Chemotherapy as Conditioning Regimen for Bone Marrow Transplantation,” Microbial Ecology 67 (2014): 690–699. 24402367 10.1007/s00248-013-0355-4

[27] J. Zwielehner , C. Lassl , B. Hippe , et al., “Changes in Human Fecal Microbiota due to Chemotherapy Analyzed by TaqMan‐PCR, 454 Sequencing and PCR‐DGGE Fingerprinting,” PLoS One 6 (2011): e28654. 22194876 10.1371/journal.pone.0028654 PMC3237468

[28] O. Youssef , L. Lahti , A. Kokkola , et al., “Stool Microbiota Composition Differs in Patients With Stomach, Colon, and Rectal Neoplasms,” Digestive Diseases and Sciences 63 (2018): 2950–2958. 29995183 10.1007/s10620-018-5190-5 PMC6182444

[29] X. Deng , Z. Li , G. Li , B. Li , X. Jin , and G. Lyu , “Comparison of Microbiota in Patients Treated by Surgery or Chemotherapy by 16S rRNA Sequencing Reveals Potential Biomarkers for Colorectal Cancer Therapy,” Frontiers in Microbiology 9 (2018): 1607. 30065719 10.3389/fmicb.2018.01607 PMC6057110

[30] A. M. Stringer , N. Al‐Dasooqi , J. M. Bowen , et al., “Biomarkers of Chemotherapy‐Induced Diarrhoea: A Clinical Study of Intestinal Microbiome Alterations, Inflammation and Circulating Matrix Metalloproteinases,” Supportive Care in Cancer 21 (2013): 1843–1852. 23397098 10.1007/s00520-013-1741-7

[31] F. Huang , S. Li , W. Chen , et al., “Postoperative Probiotics Administration Attenuates Gastrointestinal Complications and Gut Microbiota Dysbiosis Caused by Chemotherapy in Colorectal Cancer Patients,” Nutrients 15, no. 2 (2023): 356. 36678227 10.3390/nu15020356 PMC9861237

[32] S. M. Nguyen , H. T. T. Tran , J. Long , et al., “Gut Microbiome in Association With Chemotherapy‐Induced Toxicities Among Patients With Breast Cancer,” Cancer 130, no. 11 (2024): 2014–2030. 38319284 10.1002/cncr.35229

[33] H. Xinyue , X. Xuan , Z. Xiangdi , et al., “Gut Microbiota Dysbiosis Promotes the Development of Epithelial Ovarian Cancer via Regulating Hedgehog Signaling Pathway,” Gut Microbes 15, no. 1 (2023): 2221093. 37282604 10.1080/19490976.2023.2221093 PMC10249449

[34] Z. Xinlu , Z. Qi , H. Genhua , et al., “Tripterygium Glycosides Sensitizes Cisplatin Chemotherapeutic Potency by Modulating Gut Microbiota in Epithelial Ovarian Cancer,” Frontiers in Cellular and Infection Microbiology 13 (2023): 1236272. 37818040 10.3389/fcimb.2023.1236272 PMC10560985

[35] C. Jinyan , C. Xuejun , and M. Jiong , “Causal Relationships of Gut Microbiota and Blood Metabolites With Ovarian Cancer and Endometrial Cancer: A Mendelian Randomization Study,” Journal of Ovarian Research 18, no. 1 (2025): 54. 40082983 10.1186/s13048-025-01630-5 PMC11905533

[36] R. J. Wickham , “Revisiting the Physiology of Nausea and Vomiting‐Challenging the Paradigm,” Supportive Care in Cancer 28 (2020): 13–21. 31388745 10.1007/s00520-019-05012-8

[37] X. Zhao , H. Wu , R. Zhu , et al., “Combination of Thalidomide and *Clostridium butyricum* Relieves Chemotherapy‐Induced Nausea and Vomiting via Gut Microbiota and Vagus Nerve Activity Modulation,” Frontiers in Immunology 14 (2023): 1220165. 37426650 10.3389/fimmu.2023.1220165 PMC10327820

[38] X. Wang , Y. Fan , Y. Xiang , S. Zhang , and Y. Yang , “Comprehensive Gut Microbiota and Metabolomics Combined With Network Pharmacology Reveal the Effects of Acupuncture Treatment for Chemotherapy‐Induced Nausea and Vomiting,” Translational Gastroenterology and Hepatology 10 (2025): 26. 40337760 10.21037/tgh-24-35 PMC12056097

[39] X. Zhang , H. Yin , X. Yang , J. Kang , and N. Sui , “Therapeutic Mechanism of Zhuyang Tongbian Decoction in Treating Functional Constipation: Insights From a Pilot Study Utilizing 16S rRNA Sequencing, Metagenomics, and Metabolomics,” International Journal of General Medicine 18 (2025): 1007–1022. 40026814 10.2147/IJGM.S509592 PMC11871934

[40] Q. Yang , L. Zhang , Q. Li , et al., “Characterization of Microbiome and Metabolite Analyses in Patients With Metabolic Associated Fatty Liver Disease and Type II Diabetes Mellitus,” BMC Microbiology 22, no. 1 (2022): 105. 35421921 10.1186/s12866-022-02526-w PMC9011963

