# Supplementary material for: The Role of Gut Microbiota and Their Derived Metabolites in Chemotherapy‐Induced Nausea and Vomiting in Ovarian Cancer
Source: Cancer Med. 2026 Apr 2;15(4):e71752. doi: 10.1002/cam4.71752 (PMC13045238; doi:10.1002/cam4.71752)
Supplement: Supplementary file 2 — Figure S1: Microbial diversity analyses. (A) Species accumulation curve. X‐axis: Number of samples sequenced; Y‐axis: Observed species richness. The shaded area indicates 95% confidence interval. Curve plateau demonstrates adequate sampling depth. (B) Rank‐abundance distribution. Species ranked by relative abundance (high to low). Y‐axis: log2 transformed relative abundance. (C) Non‐metric multidimensional scaling (NMDS; stress = 0.15). Ordination plot showing sample clustering by group. Stress value < 0.2 indicates good ordination representation. Table S1: Results of calculating the alpha diversity of different sample species using different metric indices. Table S2: Statistical results from the fecal metabolomics data comparison between the CINV group and the non‐CINV group. Table S3:. Correlation analysis between gut microbial genera and the severity of nausea and vomiting symptoms. Table S4:. Correlation analysis between differential metabolites and the severity of nausea and vomiting symptoms. Table S5:. Changes in the feed intake of rats before and after modeling. Table S6:. Changes in body weight of rats before and after modeling. Table S7:. Changes in kaolin intake of rats before and after modeling. [file CAM4-15-e71752-s001.docx]

Supplementary Data

**1 Supplementary Materials and Methods**

***1.1 Metabolite extraction and detection***

Metabolite extraction was performed as follows [1]. (1) Sample preparation: Accurately weighed samples (approximately 50 mg each) were transferred to 2 mL centrifuge tubes. 600 µL of HPLC-grade methanol (Sigma-Aldrich, USA) containing 2-chloro-L-phenylalanine (4 ppm, Sigma-Aldrich, USA, internal standard) was added, followed by vortexing for 30 s; (2) Homogenization: Samples were homogenized with stainless steel beads using a tissue grinder (Tissuelyser-24, Shanghai Jingxin, China; 50 Hz, 120 s); (3) Sonication: Homogenized samples were sonicated at room temperature for 10 min; (4) Centrifugation and filtration: Samples were centrifuged (Centrifuge 5424R, Eppendorf, Germany; 12,000 × g, 10 min, 4°C). Supernatants were filtered through 0.22 μm membranes and transferred to LC-MS vials.

Chromatographic separation was performed on a Vanquish UHPLC system (Thermo Fisher Scientific) equipped with an ACQUITY UPLC® HSS T3 column (2.1 × 100 mm, 1.8 µm; Waters). Operational parameters. The column maintained at 40 ℃. The flow rate and injection volume were set at 0.3 mL/min and 2 μL, respectively. For LC-ESI (+)-MS analysis, the mobile phases consisted of (B2) 0.1% formic acid in acetonitrile (v/v) and (A2) 0.1% formic acid in water (v/v). Separation was conducted under the following gradient: 0~1 min, 8% B2; 1~8 min, 8%~98% B2; 8~10 min, 98% B2; 10~10.1 min, 98%~8% B2; 10.1~12 min, 8% B2. For LC-ESI (-)-MS analysis, the analytes were carried out with (B3) acetonitrile and (A3) ammonium formate (5mM). Separation was conducted under the following gradient: 0~1 min, 8% B3; 1~8 min, 8%~98% B3; 8~10 min, 98% B3; 10~10.1 min, 98%~8% B3; 10.1~12 min, 8% B3 [2].

Mass spectrum conditions were showed as follows. Mass spectrometric detection of metabolites was performed on Orbitrap Exploris 120 (Thermo Fisher Scientific, USA) with ESI ion source. Simultaneous MS1 and MS/MS (Full MS-ddMS2 mode, data-dependent MS/MS) acquisition was used. The parameters were as follows: sheath gas pressure, 40 arb; aux gas flow, 10 arb; spray voltage, 3.50 kV and -2.50 kV for ESI (+) and ESI (-), respectively; capillary temperature, 325 ℃; MS1 range, m/z 100-1000; MS1 resolving power, 60000 FWHM; number of data dependant scans per cycle, 4; MS/MS resolving power, 15000 FWHM; normalized collision energy, 30%; dynamic exclusion time, automatic [3].

***1.2 Metagenomics and Metabolomics Analysis***

***1.2.1 Sequencing Analysis of Metagenomics***

Raw sequencing reads underwent quality control and filtering prior to downstream analyses. Adapter sequences were trimmed from the raw reads using Cutadapt (version 1.2.1) [4]. Subsequently, a sliding-window approach was used to filter out reads containing low-quality regions. To minimize host-derived contamination, we aligned the processed reads against the host reference genome with BWA [5]. The resulting high-quality, host-free reads were then subjected to de novo assembly for each individual sample. Putative coding regions (CDS) were identified using MetaGeneMark [6, 7].

CDS sequences were clustered and yielded a non-redundant gene catalog [8]. The abundance of each gene within individual samples was quantified using soap.coverage [citation needed, original link is broken; replace with appropriate citation if different tool was used] based on the number of reads mapping back to the catalog. Taxonomic annotation for the non-redundant genes was assigned via the lowest common ancestor (LCA) algorithm following BLASTN alignment (e-value cutoff ＜ 0.001) against the NCBI-NT database. Functional characterization was achieved by aligning the gene sequences against several databases (GO, KEGG, EggNOG, CAZy) using the DIAMOND algorithm [9].

To identify taxa and functions exhibiting differential abundance between sample groups, Linear Discriminant Analysis Effect Size (LEfSe) analysis was applied to the annotated taxonomic and functional profiles [10]. Beta diversity that reflects compositional dissimilarities was assessed by Bray-Curtis distance metrics. These dissimilarities were visualized through Principal Coordinate Analysis (PCoA) and Non-metric Multidimensional Scaling (NMDS) [11].

***1.2.2 Metabolomics Analysis***

Metabolomics data processing and analysis were conducted sequentially. Raw LC-MS data files were first converted to mzXML format using the MSConvert tool within the Proteowizard software package (v3.0.8789) [12]. Subsequent peak detection, filtering, and alignment were performed using the XCMS R package, with parameter settings as follows: bw = 2, ppm = 15, peakwidth = c(5, 30), mzwid = 0.015, mzdiff = 0.01, and method = "centWave", resulting in a compound quantification table.

Metabolite annotation was carried out by comparing the acquired MS/MS spectra of metabolites in the quantitative list against reference spectral libraries, including HMDB, MassBank, LipidMaps, mzCloud, and KEGG. Structural characterization was achieved by matching fragment ion information between the experimental spectra and reference databases, corresponding to Confidence Level 2 annotation.

Prior to statistical analysis, the data matrix underwent quality control and preprocessing. Systemic errors were corrected using support vector regression (SVR) normalization based on quality control (QC) samples. To ensure data reliability, metabolites exhibiting a coefficient of variance (CV) greater than 30% in QC samples were excluded. Finally, the dataset was normalized using a QC-based normalization approach. Following these preprocessing steps, both univariate and multivariate statistical analyses were performed as described in the main text. Finally, pathway enrichment analysis of the differentially expressed metabolites was performed using MetaboAnalyst [13].

***1.3 Immunohistochemistry***

Formalin-fixed, paraffin-embedded (FFPE) tissue blocks were sectioned at 4 µm thickness and mounted on poly-L-lysine-coated slides. Sections were baked at 60°C for 2 h, deparaffinized in xylene (2 × 10 min), and rehydrated through a graded ethanol series (100%, 95%, 85%, 75%; 5 min per concentration) followed by rinsing in deionized water. Antigen retrieval was performed by microwave heating in Tris-EDTA buffer (10 mM Tris, 1 mM EDTA, pH 9.0) for 20 min. Endogenous peroxidase activity was quenched by incubation with 3% H₂O₂ for 10 min. Sections were incubated with primary antibodies for 1 h at room temperature, followed by HRP-conjugated anti-rabbit secondary antibodies (1:200 dilution) for 30 min. Immunoreactivity was visualized using 3,3′-diaminobenzidine (DAB) chromogen for 8 min, and nuclei were counterstained with Mayer’s hematoxylin for 60 s. Appropriate positive and negative controls were also prepared to assess the quality control of this immunohistochemical staining protocol. Antibodies included rabbit polyclonal antibodies in details were shown below.

***Antibodies used in IHC***

| **Antibody** | **Company** | **Stata** | **Product No.** | **Dilution** |
| --- | --- | --- | --- | --- |
| 5-HT3R | Abcam | UK | ab13897 | 1:200 |
| NK1R | Abcam | UK | ab317504 | 1:150 |
| NK2R | Abcam | UK | ab231716 | 1:150 |

***1.4 Primers used in SD rat***

| **Gene** | **Sense** | **Anti-sense** | **Product size** |
| --- | --- | --- | --- |
| 5-HT3R | TCTGGGGGACTCATCCTG | TGGGCTATCAGCACGGAAA | 118 |
| NK1R | CTGACCGCTACCATGAGCAA | CAGCAGATGGCGAAGGTACA | 85 |
| NK2R | CCTGCTTGGTGGTCACTCAT | GTACTGGCGCAGTGAACTCT | 120 |
| GAPDH | AACAGCCTCAAGATCATCAGC | ATGAGTCCTTCCACGATACCAA | 101 |

***1.5 Antibodies used in Western blotting***

| **Antibody** | **Company** | **Stata** | **Product No.** | **Dilution** |
| --- | --- | --- | --- | --- |
| 5-HT3R | Abcam | UK | ab13897 | 1:2000 |
| NK1R | Proteintech | USA | 17942-1-AP | 1:600 |
| NK2R | Abcam | UK | ab231716 | 1:2000 |
| GAPDH | Proteintech | USA | 10494-1-AP | 1:20000 |

**2 Supplementary Figures**


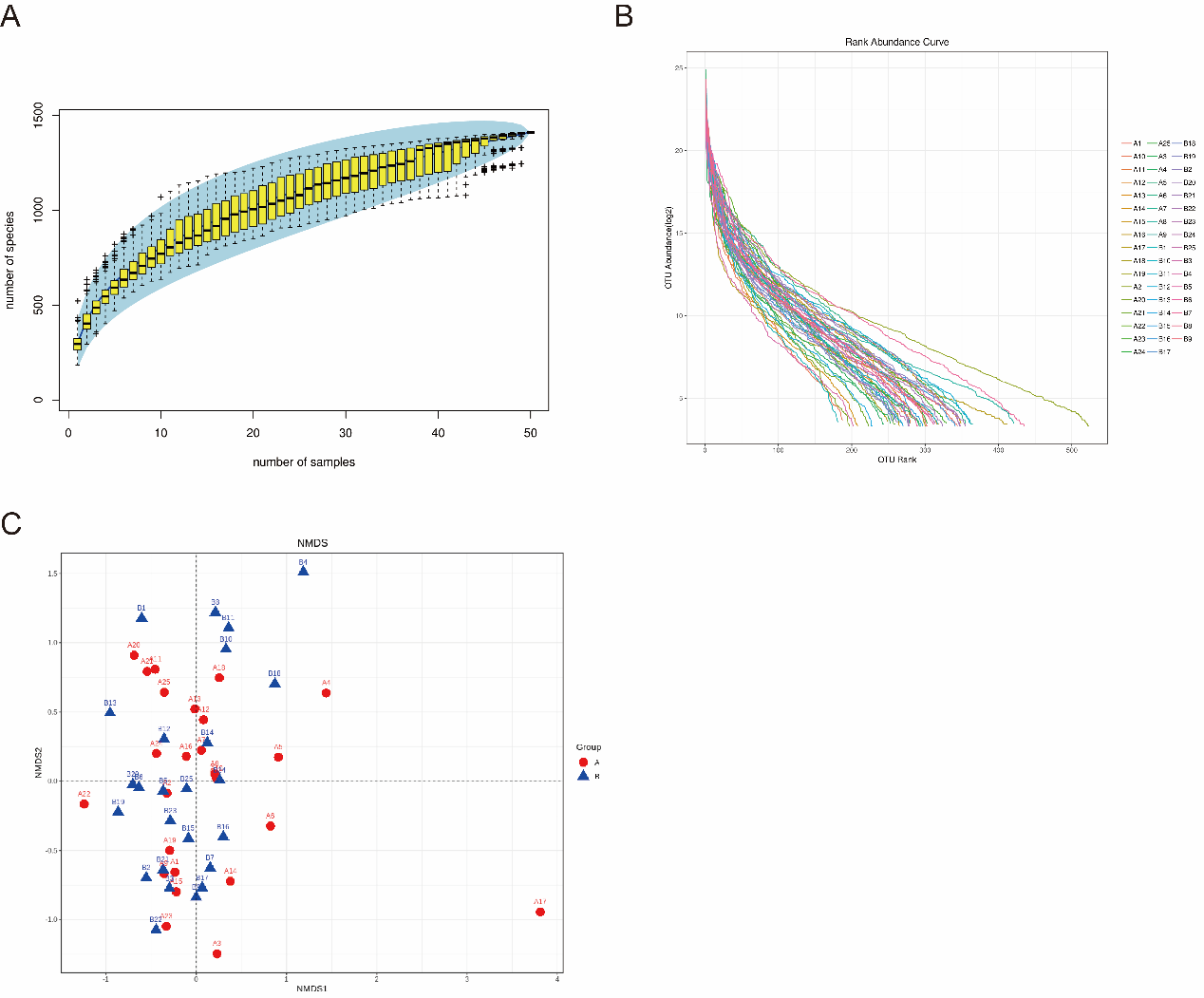


**Figure S1.** Microbial diversity analyses. (A) Species accumulation curve. X-axis: Number of samples sequenced; Y-axis: Observed species richness. Shaded area indicates 95% confidence interval. Curve plateau demonstrates adequate sampling depth. (B) Rank-abundance distribution. Species ranked by relative abundance (high to low). Y-axis: log2 transformed relative abundance. (C) Non-metric multidimensional scaling (NMDS; stress = 0.15). Ordination plot showing sample clustering by group. Stress value <0.2 indicates good ordination representation.

**3 Supplementary Tables**

**Table S1.** Results of calculating the alpha diversity of different sample species using different metric indices

| **Samples** | **simpson** | **chao1** | **ace** | **shannon** |
| --- | --- | --- | --- | --- |
| A1 | 0.9093495322176 | 344.0 | 344.0 | 4.269033248901135 |
| A2 | 0.913728729079163 | 523.0 | 523.0 | 4.713290785667732 |
| A3 | 0.5432330702035395 | 253.0 | 253.0 | 2.06097197583743 |
| A4 | 0.2078391708237708 | 244.0 | 244.0 | 0.9845478512372481 |
| A5 | 0.7840840672258396 | 294.0 | 294.0 | 3.378796081355592 |
| A6 | 0.8942181493357252 | 355.0 | 355.0 | 4.411370813190827 |
| A7 | 0.8987143148768774 | 268.0 | 268.0 | 3.99594418142102 |
| A8 | 0.9071349519726853 | 421.0 | 421.0 | 4.336452856769177 |
| A9 | 0.8864310570113771 | 365.0 | 365.0 | 4.362931376215054 |
| A10 | 0.626602182485743 | 188.0 | 188.0 | 2.334208709095885 |
| A11 | 0.8497104105202942 | 280.0 | 280.0 | 3.4999009780697747 |
| A12 | 0.8271864431515175 | 281.0 | 281.0 | 3.622040896534533 |
| A13 | 0.8394330107632679 | 319.0 | 319.0 | 3.495062968524947 |
| A14 | 0.5699346343825664 | 208.0 | 208.0 | 2.1881876301995558 |
| A15 | 0.8982152342555529 | 349.0 | 349.0 | 4.511694626324113 |
| A16 | 0.9006536966646118 | 265.0 | 265.0 | 4.07170730219894 |
| A17 | 0.748287505128592 | 412.0 | 412.0 | 2.7771131033813115 |
| A18 | 0.7812440362979001 | 249.0 | 249.0 | 3.281134669320926 |
| A19 | 0.8085098458784289 | 301.0 | 301.0 | 3.9046186048267653 |
| A20 | 0.8723525469417509 | 197.0 | 197.0 | 3.8531100977391164 |
| A21 | 0.8582106154433282 | 223.0 | 223.0 | 3.4505044123855257 |
| A22 | 0.9256887208095189 | 260.0 | 260.0 | 4.5894176669104665 |
| A23 | 0.8379129522263761 | 329.0 | 329.0 | 3.9938588828042954 |
| A24 | 0.9245734385271243 | 303.0 | 303.0 | 4.605418528964577 |
| A25 | 0.8446326006590368 | 294.0 | 294.0 | 3.4914321437901603 |
| B1 | 0.78437811954573 | 182.0 | 182.0 | 3.0586783844643515 |
| B2 | 0.93171386435558 | 347.0 | 347.0 | 4.680125181584238 |
| B3 | 0.8739127416455712 | 278.0 | 278.0 | 3.9216316022627957 |
| B4 | 0.4424092871561818 | 202.0 | 202.0 | 1.6216389920541432 |
| B5 | 0.9150897230688183 | 312.0 | 312.0 | 4.304951673616458 |
| B6 | 0.9529753169850709 | 290.0 | 290.0 | 4.957394310103279 |
| B7 | 0.9287362989776997 | 435.0 | 435.0 | 4.813292870742304 |
| B8 | 0.7129670218302453 | 308.0 | 308.0 | 2.69249572919348 |
| B9 | 0.8272008333420829 | 303.0 | 303.0 | 3.468077906323754 |
| B10 | 0.7711136771677028 | 318.0 | 318.0 | 3.0137400680298203 |
| B11 | 0.6905490215226329 | 279.0 | 279.0 | 2.977918169927592 |
| B12 | 0.9367195389372879 | 361.0 | 361.0 | 4.752832044686799 |
| B13 | 0.7491447263556955 | 228.0 | 228.0 | 2.7977649363908395 |
| B14 | 0.7038522051267284 | 270.0 | 270.0 | 2.886060031160756 |
| B15 | 0.8670851900510398 | 297.0 | 297.0 | 3.8173413798608085 |
| B16 | 0.9425430666910419 | 341.0 | 341.0 | 4.7516692382231005 |
| B17 | 0.7916514142200208 | 258.0 | 258.0 | 3.2146466209792424 |
| B18 | 0.8995987703923789 | 256.0 | 256.0 | 3.908297916169201 |
| B19 | 0.886445614406438 | 277.0 | 277.0 | 3.93184149102833 |
| B20 | 0.8254768798928377 | 263.0 | 263.0 | 3.4565484932084702 |
| B21 | 0.9171097840781267 | 324.0 | 324.0 | 4.461647669412715 |
| B22 | 0.9426729348010187 | 355.0 | 355.0 | 4.948828427147597 |
| B23 | 0.9349821916540488 | 314.0 | 314.0 | 4.9239667371541405 |
| B24 | 0.7015682524925583 | 348.0 | 348.0 | 2.7555913603715676 |
| B25 | 0.8161298735932957 | 274.0 | 274.0 | 3.3152250565463595 |

The first column in the table is the sample name, and the following four columns are the calculated results of the four diversity indices for each sample.

**Table S2.** Statistical results from the fecal metabolomics data comparison between the CINV group and the non-CINV group

| **Metabolite** | **CAS Number** | **FC (CINV vs. Non-CINV)** | **log2FC** | **P** |
| --- | --- | --- | --- | --- |
| Myristoleic acid | 544-64-9 | 7.65 | 2.94 | 0.040903 |
| Glycitein | 40957-83-3 | 4.72 | 2.24 | 0.035282 |
| Sinapyl alcohol | 537-33-7 | 3.47 | 1.8 | 0.049096 |
| Dimethylglycine | 1118-68-9 | 2.82 | 1.5 | 0.029503 |
| Gluconolactone | 90-80-2 | 2.73 | 1.45 | 0.028247 |
| N-Carbamoylputrescine | 6851-51-0 | 2.61 | 1.38 | 0.002708 |
| Hesperetin | 520-33-2 | 2.55 | 1.35 | 0.004089 |
| Pregnanediol | 80-92-2 | 2.45 | 1.29 | 0.020009 |
| gamma-Glutamylcysteine | 636-58-8 | 2.37 | 1.24 | 0.000616 |
| alpha-Ketoisovaleric acid | 759-05-7 | 2.31 | 1.21 | 0.000316 |
| Malonate | 141-82-2 | 2.29 | 1.2 | 0.012199 |
| 3'-Ketolactose | 15990-62-2 | 2.17 | 1.12 | 7.66E-10 |
| Lovastatin | 75330-75-5 | 2.03 | 1.02 | 0.02557 |
| Orciprenaline | 586-06-1 | 1.93 | 0.95 | 0.00814 |
| 7-Dehydrocholesterol | 434-16-2 | 1.84 | 0.88 | 0.047135 |
| 6-Hydroxynicotinic acid | 5006-66-6 | 1.79 | 0.84 | 0.023672 |
| Oxoglutaric acid | 328-50-7 | 1.65 | 0.73 | 0.018791 |
| Arbutin | 497-76-7 | 1.6 | 0.68 | 0.039251 |
| Betulin | 472-15-1 | 1.52 | 0.6 | 0.026222 |
| Catechol | 120-80-9 | 0.66 | -0.6 | 0.009806 |
| Pyroglutamic acid | 98-79-3 | 0.66 | -0.61 | 0.034999 |
| Mesaconate | 498-24-8 | 0.65 | -0.63 | 0.000914 |
| Betulinic acid | 472-15-1 | 0.62 | -0.68 | 0.038817 |
| 7-Oxodeoxycholate | 911-40-0 | 0.49 | -1.03 | 0.005636 |
| 2-Deoxystreptamine | 2037-48-1 | 0.47 | -1.09 | 0.015953 |
| Uracil 5-carboxylate | 23945-44-0 | 0.36 | -1.46 | 0.027037 |
| D-Ribose 5-phosphate | 4300-28-1 | 0.32 | -1.67 | 0.039549 |
| 4,5,6,7-Tetrahydroisoxazolo(5,4-c)pyridin-3-ol | 64603-91-4 | 0.29 | -1.78 | 0.014132 |
| 2-Hydroxyisophthalic acid | 606-19-9 | 0.22 | -2.2 | 2.16E-09 |

FC, Fold Change.

**Table S3.** Correlation analysis between gut microbial genera and the severity of nausea and vomiting symptoms

|  | **Nausea** | | **Vomiting** | | |
| --- | --- | --- | --- | --- | --- |
| **Intestinal flora** | **R** | **P** | | **R** | **P** |
| g__Bacteroides | 0.691 | **＜0.001** | | 0.571 | **0.003** |
| g__Blautia | 0.272 | 0.189 | | 0.109 | 0.606 |
| g__Bifidobacterium | ＜0.001 | 1.000 | | -0.152 | 0.468 |
| g__Mediterraneibacter | 0.170 | 0.417 | | 0.080 | 0.705 |
| g__Enterococcus | -0.532 | **0.006** | | -0.334 | 0.102 |
| g__Streptococcus | -0.306 | 0.137 | | -0.260 | 0.210 |
| g__Parabacteroides | 0.696 | **＜0.001** | | 0.594 | **0.002** |
| g__Escherichia | -0.136 | 0.517 | | -0.072 | 0.732 |
| g__Faecalibacterium | 0.340 | 0.097 | | 0.396 | 0.050 |
| g__Erysipelatoclostridium | 0.023 | 0.914 | | -0.130 | 0.537 |
| g__Phocaeicola | 0.442 | **0.027** | | 0.451 | **0.024** |
| g__Eggerthella | 0.260 | 0.209 | | 0.176 | 0.400 |
| g__Collinsella | 0.034 | 0.872 | | 0.079 | 0.707 |
| g__Dorea | 0.079 | 0.706 | | 0.093 | 0.659 |
| g__Klebsiella | 0.147 | 0.483 | | 0.109 | 0.605 |
| g__Anaerostipes | 0.283 | 0.170 | | 0.136 | 0.515 |
| g__Anaerobutyricum | -0.045 | 0.830 | | 0.039 | 0.854 |
| g__Akkermansia | 0.471 | **0.018** | | 0.332 | 0.105 |
| g__Citrobacter | -0.215 | 0.302 | | -0.202 | 0.334 |
| g__Ruthenibacterium | 0.419 | **0.037** | | 0.325 | 0.113 |

The prefix "g_" denotes bacterial genus. The correlation analysis was performed by non parametric Spearman method. R represents the Spearman correlation coefficient. Only genera with significant correlations (P < 0.05) in the initial screening are shown for clarity.

**Table S4.** Correlation analysis between differential metabolites and the severity of nausea and vomiting symptoms

|  | **Nausea** | | **Vomiting** | | |
| --- | --- | --- | --- | --- | --- |
| **Metabolite** | **R** | **P** | | **R** | **P** |
| Dimethylglycine | 0.159 | 0.270 | | 0.182 | 0.205 |
| Catechol | -0.355 | **0.011** | | -0.352 | **0.012** |
| N-Carbamoylputrescine | 0.255 | 0.074 | | 0.191 | 0.185 |
| 6-Hydroxynicotinic acid | 0.309 | **0.029** | | 0.218 | 0.128 |
| 4,5,6,7-Tetrahydroisoxazolo(5,4-c)pyridin-3-ol | -0.248 | 0.082 | | -0.176 | 0.222 |
| 2-Deoxystreptamine | -0.296 | **0.037** | | -0.275 | 0.053 |
| Orciprenaline | 0.411 | **0.003** | | 0.324 | **0.022** |
| Myristoleic acid | 0.328 | **0.020** | | 0.292 | **0.040** |
| D-Ribose 5-phosphate | -0.185 | 0.199 | | -0.183 | 0.203 |
| Hesperetin | 0.386 | **0.006** | | 0.322 | **0.023** |
| Pregnanediol | 0.186 | 0.197 | | 0.101 | 0.486 |
| 7-Dehydrocholesterol | 0.304 | **0.032** | | 0.217 | 0.130 |
| Lovastatin | 0.174 | 0.226 | | 0.065 | 0.625 |
| Betulin | 0.407 | **0.003** | | 0.395 | **0.004** |
| Betulinic acid | -0.272 | 0.056 | | -0.257 | 0.072 |
| Malonate | 0.188 | 0.192 | | 0.130 | 0.367 |
| alpha-Ketoisovaleric acid | 0.552 | **＜0.001** | | 0.502 | **＜0.001** |
| Pyroglutamic acid | -0.251 | 0.078 | | -0.231 | 0.106 |
| Mesaconate | -0.386 | **0.006** | | -0.440 | **0.001** |
| Oxoglutaric acid | 0.310 | **0.029** | | 0.322 | **0.023** |
| Uracil 5-carboxylate | -0.337 | **0.017** | | -0.214 | 0.136 |
| Gluconolactone | 0.185 | 0.199 | | 0.167 | 0.247 |
| 2-Hydroxyisophthalic acid | 0.658 | **＜0.001** | | -0.631 | **＜0.001** |
| Sinapyl alcohol | 0.155 | 0.281 | | 0.165 | 0.253 |
| gamma-Glutamylcysteine | 0.238 | 0.096 | | 0.161 | 0.264 |
| Arbutin | 0.399 | **0.004** | | 0.334 | **0.018** |
| Glycitein | 0.254 | 0.075 | | 0.247 | 0.084 |
| 3'-Ketolactose | 0.748 | **＜0.001** | | 0.687 | **＜0.001** |
| 7-Oxodeoxycholate | -0.372 | **0.008** | | -0.315 | **0.026** |

The correlation analysis was performed by non parametric Spearman method. R represents the Spearman correlation coefficient.

**Table S5.** Changes in feed intake of rats before and after modeling

| **Time** | **Control** | **Cisplatin** | **Non-CINV microbiota transplantation cisplatin** | **CINV microbiota transplantation cisplatin** | **P** |
| --- | --- | --- | --- | --- | --- |
| -48h | 20.357±1.076 | 20.237±1.220 | 20.106±1.000 | 20.814±1.126 | 0.517 |
| -24h | 20.444±1.100 | 20.414±1.205 | 20.363±1.065 | 20.700±1.309 | 0.920 |
| 0h | 20.453±0.988 | 20.402±1.351 | 20.296±0.938 | 20.443±1.171 | 0.989 |
| 24h | 20.581±0.937 | 10.312±1.079 | 18.035±1.805 | 19.931±1.390 | ＜0.001 |
| 48h | 20.467±1.037 | 8.341±1.737 | 16.264±1.567 | 8.551±0.972 | ＜0.001 |
| 72h | 20.389±1.092 | 7.634±1.174 | 14.704±1.234 | 8.527±0.982 | ＜0.001 |

CINV, chemotherapy-induced nausea and vomiting.

**Table S6.** Changes in body weight of rats before and after modeling

| **Time** | **Control** | **Cisplatin** | **Non-CINV microbiota transplantation cisplatin** | **CINV microbiota transplantation cisplatin** | **P** |
| --- | --- | --- | --- | --- | --- |
| -48h | 189.105±4.025 | 189.258±3.704 | 189.774±2.935 | 189.658±3.345 | 0.970 |
| -24h | 196.344±3.873 | 196.328±4.054 | 197.101±3.734 | 196.572±3.705 | 0.966 |
| 0h | 202.906±3.860 | 202.606±3.908 | 203.809±4.182 | 203.061±3.822 | 0.917 |
| 24h | 209.090±3.932 | 192.523±3.034 | 198.899±4.478 | 194.966±3.902 | ＜0.001 |
| 48h | 214.437±4.257 | 183.019±3.399 | 189.997±4.564 | 187.444±4.535 | ＜0.001 |
| 72h | 219.546±4.341 | 175.896±3.368 | 185.099±5.628 | 178.624±4.523 | ＜0.001 |

CINV, chemotherapy-induced nausea and vomiting.

**Table S7.** Changes in kaolin intake of rats before and after modeling

| **Time** | **Control** | **Cisplatin** | **Non-CINV microbiota transplantation cisplatin** | **CINV microbiota transplantation cisplatin** | **P** |
| --- | --- | --- | --- | --- | --- |
| -48h | 0.446±0.171 | 0.465±0.143 | 0.488±0.136 | 0.465±0.136 | 0.938 |
| -24h | 0.432±0.140 | 0.409±0.113 | 0.485±0.121 | 0.512±0.141 | 0.281 |
| 0h | 0.122±0.071 | 0.118±0.096 | 0.116±0.094 | 0.128±0.094 | 0.991 |
| 24h | 0.136±0.053 | 1.295±0.154 | 0.780±0.138 | 1.078±0.124 | ＜0.001 |
| 48h | 0.266±0.079 | 1.244±0.269 | 0.578±0.134 | 1.063±0.155 | ＜0.001 |
| 72h | 0.207±0.075 | 0.936±0.231 | 0.622±0.135 | 0.975±0.083 | ＜0.001 |

CINV, chemotherapy-induced nausea and vomiting.

**Reference**

[1] Turroni S, Fiori J, Rampelli S, Schnorr SL, Consolandi C, Barone M, et al. Fecal metabolome of the Hadza hunter-gatherers: a host-microbiome integrative view. Sci Rep. 2016;6:32826.

[2] Zelena E, Dunn WB, Broadhurst D, Francis-McIntyre S, Carroll KM, Begley P, et al. Development of a robust and repeatable UPLC-MS method for the long-term metabolomic study of human serum. Anal Chem. 2009;81:1357-64.

[3] Want EJ, Masson P, Michopoulos F, Wilson ID, Theodoridis G, Plumb RS, et al. Global metabolic profiling of animal and human tissues via UPLC-MS. Nat Protoc. 2013;8:17-32.

[4] Martin M. Cutadapt removes adapter sequences from high-throughput sequencing reads. EMBnetjournal. 2011;17:10-2.

[5] Li H, Durbin R. Fast and accurate short read alignment with Burrows–Wheeler transform. Bioinformatics. 2009;25:1754-60.

[6] Peng Y, Leung HCM, Yiu SM, Chin FYL. IDBA-UD: a de novo assembler for single-cell and metagenomic sequencing data with highly uneven depth. Bioinformatics. 2012;28:1420-8.

[7] Zhu W, Lomsadze A, Borodovsky M. Ab initio gene identification in metagenomic sequences. Nucleic Acids Research. 2010;38:e132-e.

[8] Fu L, Niu B, Zhu Z, Wu S, Li W. CD-HIT: accelerated for clustering the next-generation sequencing data. Bioinformatics. 2012;28:3150-2.

[9] Benjamin B, Chao X, Daniel H H. Fast and sensitive protein alignment using DIAMOND. Nat Methods. 2014;12.

[10] Nicola S, Jacques I, Levi W, Dirk G, Larisa M, Wendy S G, et al. Metagenomic biomarker discovery and explanation. Genome Biol. 2011;12.

[11] Alban R. Multivariate analyses in microbial ecology. FEMS Microbiol Ecol. 2007;62.

[12] Thevenot EA, Roux A, Xu Y, Ezan E, Junot C. Analysis of the Human Adult Urinary Metabolome Variations with Age, Body Mass Index, and Gender by Implementing a Comprehensive Workflow for Univariate and OPLS Statistical Analyses. J Proteome Res. 2015;14:3322-35.

[13] Xia J, Wishart DS. Web-based inference of biological patterns, functions and pathways from metabolomic data using MetaboAnalyst. Nat Protoc. 2011;6:743-60.
